# Cryoprotectant-Mediated Cold Stress Mitigation in Litchi Flower Development: Transcriptomic and Metabolomic Perspectives

**DOI:** 10.3390/metabo14040223

**Published:** 2024-04-15

**Authors:** Xue-Wen Zheng, Xin-Yue Cao, Wen-Hao Jiang, Guang-Zhao Xu, Qing-Zhi Liang, Zhuan-Ying Yang

**Affiliations:** College of Coastal Agricultural Sciences, Guangdong Ocean University, Zhanjiang 524088, China; 2112104035@stu.gdou.edu.cn (X.-W.Z.); 202111321102@stu.gdou.edu.cn (X.-Y.C.); 2112204052@stu.gdou.edu.cn (W.-H.J.); xugz@gdou.edu.cn (G.-Z.X.); qingzhi2002@gdou.edu.cn (Q.-Z.L.)

**Keywords:** plant growth regulator, low temperature, Bihu, Liangli, β-alanine

## Abstract

Temperature is vital in plant growth and agricultural fruit production. *Litchi chinensis* Sonn, commonly known as litchi, is appreciated for its delicious fruit and fragrant blossoms and is susceptible to stress when exposed to low temperatures. This study investigates the effect of two cryoprotectants that counteract cold stress during litchi flowering, identifies the genes that generate the cold resistance induced by the treatments, and hypothesizes the roles of these genes in cold resistance. Whole plants were treated with Bihu and Liangli cryoprotectant solutions to protect inflorescences below 10 °C. The soluble protein, sugar, fructose, sucrose, glucose, and proline contents were measured during inflorescence. Sucrose synthetase, sucrose phosphate synthetase, antioxidant enzymes (SOD, POD, CAT), and MDA were also monitored throughout the flowering stage. Differentially expressed genes (DEGs), gene ontology, and associated KEGG pathways in the transcriptomics study were investigated. There were 1243 DEGs expressed after Bihu treatment and 1340 in the control samples. Signal transduction pathways were associated with 39 genes in the control group and 43 genes in the Bihu treatment group. The discovery of these genes may contribute to further research on cold resistance mechanisms in litchi. The Bihu treatment was related to 422 low-temperature-sensitive differentially accumulated metabolites (DAMs), as opposed to 408 DAMs in the control, mostly associated with lipid metabolism, organic oxidants, and alcohols. Among them, the most significant differentially accumulated metabolites were involved in pathways such as β-alanine metabolism, polycyclic aromatic hydrocarbon biosynthesis, linoleic acid metabolism, and histidine metabolism. These results showed that Bihu treatment could potentially promote these favorable traits and increase fruit productivity compared to the Liangli and control treatments. More genomic research into cold stress is needed to support the findings of this study.

## 1. Introduction

Litchi (*Litchi chinensis* Sonn.) is a tropical fruit distributed broadly within the subtropic regions of southern China, Malaysia, and northern Vietnam. China generates more than half of the world’s litchi supply, accounting for more than 60% of worldwide litchi production [1]. Litchi flowering requires a low temperature [2]. However, rapid freezing during flowering results in the premature appearance of inflorescence [3]. Seasonal and regional variations in climate impact several growth and development processes and agricultural productivity [4]. The annual litchi harvest is primarily determined by a successful initiation of the flowers, influenced by several external and natural cues, soil moisture levels, and temperature fluctuations [5]. The global climate change is anticipated to adversely impact flower development and the initiation of floral expansion [6]. After inflorescence initiation, litchi requires a temperature above 15 °C for growth [7]. If the temperature drops below 10 °C during this period, the litchi flower can suffer from cold stress, which leads to pollen and floral organ injury and affects the normal flowering and fruit setting [8]. While ambient temperatures above 20 °C considerably decrease litchi flowering, exposure to cold temperatures in winter or early spring accelerates the start of litchi floral growth [3]. Unusually high winter temperatures can prevent achieving the cold conditions required to initiate the litchi floral cycle, resulting in insufficient flowering [9]. According to previous research, drought before winter cold can, to some extent, encourage litchi flowering by lessening the need for cold temperatures [10,11]. 

Low temperature is a major abiotic stress that causes the production of reactive oxygen species (ROS), which can harm numerous plant structures, agricultural crop growth, productivity, and plant geographical distribution, leading to significant crop loss [12,13]. Increased ROS production can be dangerous by causing damage to proteins and DNA, blocking important enzymes, and leading to cell death [14]. The balance between ROS production and the scavenging capacities of the fruit largely determines ROS accumulation. A plant’s ROS amount is usually controlled by specific enzymes like superoxide dismutases, catalases, and peroxidases [15]. Plants have developed various mechanisms to cope with adverse conditions such as low temperatures [16,17]. This process involves several biochemical and physiological changes, including increased levels of proline, soluble sugars, and enzyme activities, as well as a decreased level of MDA [16,17]. Cold-tolerant cultivars present the capacity to accumulate sucrose and proline [17,18]. When polyphenols are exposed to peroxidase and hydrogen peroxide, they can turn the outer layer of the litchi fruit brown. This process helps eliminate harmful ROS [19]. In alfalfa, root nodules may improve alfalfa’s ability to survive low temperatures by enhancing the activity of antioxidant enzymes and influencing the expression of metabolism-related genes like those involved in soluble protein and sugar accumulation [20]. Adding brassinosteroid (BR) to alfalfa seeds increased the activity of antioxidant enzymes. 

Guangdong is the province in China with the greatest litchi cultivation and production [21]. It has a tropical and subtropical monsoon climate, with an average annual temperature ranging from 20 °C to 28 °C [22]. However, during the winter and spring seasons, there are often several consecutive days of low temperatures or frost, with temperatures possibly dropping below 10 °C. This cold weather significantly affects the development of litchi inflorescences. However, cold treatment alone does induce litchi flowering. The objective of the present study was to investigate the impact of low temperatures on safeguarding inflorescences after floral initiation in potted litchi plants. Next-generation RNA sequencing was used to acquire an overview of the transcriptome modifications during inflorescence induction. The expression profiles of unigenes after different treatments were compared and characterized to discover potentially relevant genes involved in litchi floral initiation in response to drought and cold.

There is limited research on litchi inflorescence growth and development in the presence of low-temperature stress, and the related molecular mechanisms are still unclear. This study conducted pre-treatments with Bihu (BF) and Liangli (LL) (plant growth regulators, PGRs) before the occurrence of low temperatures. By measuring physiological and biochemical indicators and performing transcriptomics and metabolomics analyses, the preliminary cold resistance mechanism of litchi inflorescences was explored, providing crucial theoretical support for mitigating low-temperature damage during litchi production. This study aimed to enhance litchi inflorescence growth and development under low-temperature stress through the environmentally friendly Bihu and Liangli treatments, as well as to identify the genes responsible for the cold resistance conferred by these treatments and hypothesize their role. Transcriptase and metabolome analyses may provide helpful insights into the molecular mechanisms of litchi inflorescence development under low-temperature stress.

## 2. Materials and Methods

### 2.1. Plant Materials and Experimental Design

During the flowering season from January to March 2022, the experiments were conducted on 12 20-year-old Feizixiao litchi varieties with similar phenological stages and flowering periods in Longxiang Orchard, Yangxi County, Yangjiang City, Guangdong Province (long. 111°45″ E, lat. 21°66″ N). The randomly selected Feizixiao plants were divided into three groups. Then, on 17 February 2022, the plants were sprayed with a Bihu solution (chemical composition: 0.135% gibberellic acid, 0.00052% indole acetic acid, and 0.00031% brassinolide; BF group), a Liangli solution (chemical composition: 0.5 mL/L of 1,1-dimethylpiperidine chloride; LL group), or clean distilled water (control, CK group). These treatments were performed according to the weather forecast, two days before the onset of low temperatures (average temperature ≤10 °C), and each treatment was performed four times. Flower spike samples were collected 0 d, 2 d, 3 d, 4 d, 5 d, 8 d, and 14 d after treatment, immediately frozen in liquid nitrogen, and then stored at −40 °C for further analysis. The biochemical analysis was conducted in the Tropical Crop Efficient Production Engineering Technology Research Center, Binhai Agricultural College, Guangdong Ocean University. Each treatment was performed in three biological replicates. 

### 2.2. Biochemical Characterization of the Flower Spikes

#### 2.2.1. Determination of Soluble Protein and Sugars

Total soluble protein was determined using the Coomassie brilliant blue method according to [23], and the sugar content was determined by using HPLC (high-performance liquid chromatography) assays according to Medlicott, A. P. et al. [24]. For soluble protein determination, flower spike samples homogenized in 50 mM Tris–HCl, 2 mM EDTA (ethylenediamine tetraacetic acid), pH 7.5, and 0.04% (*v*/*v*) 2-mercaptoethanol were centrifuged at 12,000× *g* for 15 min at 25 °C. The supernatant was mixed with 1 mL of CBB (Coomassie brilliant blue), and the optical density was determined at 595 nm using a spectrophotometer. For soluble sugar determination, 2 g of flower spike samples was homogenized with 3 mL of UP water and placed in a microwave oven for 40 s. Then, the homogenized samples were centrifuged at 12,000× *g* for 5 min, and the supernatants were used to detect soluble sugar. The chromatographic conditions included an Agela Technologies NH_2_ column (4.6 mm × 250 mm, 5 μm), a column temperature of 40 °C, a mobile phase of acetonitrile/water, 8:2 *v/v*, and a flow rate of 1.0 mL min^−1^; an RID detector was used.

#### 2.2.2. Estimation of Malondialdehyde (MDA) and Proline

The content of malondialdehyde (MDA) was determined according to Dhindsa, R.S. et al. [25], and that of proline with the acidic ninhydrin method [26]. For MDA determination, 25 g of the flower bud samples was homogenized in 5 mL of 0.1% TCA and centrifuged at 10,000× *g* for 5 min. Then, a 1 mL aliquot of the supernatant was combined with 4 mL of 20% trichloroacetic acid (TCA) containing 0.5% thiobarbituric acid (TBA). The resulting mixture was then heated at 95 °C for 30 min, followed by rapid cooling in an ice bath. Again, the samples were centrifuged at 10,000× *g* for 10 min, their optical density was measured at 532 nm, and the value of non-specific absorption at 600 nm was subtracted from the measured values. The concentration of MDA was calculated by applying its extinction coefficient of 155 mM cm^−1^. For soluble proline analysis, flower bud samples were ground using a 3% (*w*/*v*) sulfosalicylic acid aqueous solution. The resulting homogenates were filtered through Whatman No. 1 filter paper. Following filtration, 2 mL of the filtered extracts was subjected to further analysis. To this purpose, 2 mL of acid ninhydrin and 2 mL of glacial acetic acid were added to each extract. The resulting reaction mixtures were incubated in a boiling water bath for 1 h, and the reaction was terminated in an ice bath. To extract the reaction product, 4 mL of toluene was added to the mixtures. The organic phase containing the product was separated, and its absorbance was measured at 520 nm using a UV–visible spectrophotometer (UV-5100B, Shanghai Xiwen Biotech. Co., Ltd., Jia’An Road, Jiading District, Shanghai, China); toluene was used as a blank.

#### 2.2.3. Analysis of Antioxidant Enzymes (CAT, SOD, and POD)

The activities of the antioxidant enzymes superoxide dismutase (SOD), peroxidase (POD), and catalase (CAT) were measured according to Shah, K. et al., Sun, M. et al., and Verma, S. et al. [27,28,29], respectively. Each sample of flower buds (0.2 g) was individually crushed in 10 mL of phosphate buffer (100 mM, pH 7.0) to evaluate the different antioxidant activities. Subsequently, the homogenized samples were centrifugated at 4 °C and 13,000× *g* for 10 min, and the supernatants were collected. For CAT activity evaluation, reaction mixtures comprising 1.5 mL of potassium phosphate buffer (100 mM, pH 7.0), 400 μL of H_2_O_2_ (200 mM), and 100 μL of leaf extract were prepared. The reduction in absorbance at 240 nm was then recorded using a UV–vis spectrophotometer. As for SOD, a reaction was set up by combining 1.3 mL of sodium bicarbonate or carbonate buffer (50 mM, pH 9.8), 100 µL of EDTA (0.1 mM), and 500 µL of epinephrine (0.6 mM). Subsequently, adrenochrome formation was measured using a spectrophotometer at 475 nm. Further, POD activity was investigated in a reaction mixture containing 100 mM potassium phosphate buffer (pH 6.5). The components included 200 μL of 16 mM guaiacol, 20 μL of 6% H_2_O_2_, and 100 μL of the flower bud extract. The increase in absorbance was monitored at 470 nm using a UV–vis spectrophotometer at 10 s intervals for up to 1 min, and enzyme activity is expressed as nmol^−1^(mg protein)^−1^.

#### 2.2.4. Estimation of the Activities of Key Proline Enzymes (P5CS, P5CR, ProDH, and δ-OAT)

The activities of the key proline enzymes pyrroline-5-carboxylate synthase (P5CS), pyrroline-5-carboxylate reductase (P5CR), proline dehydrogenase (ProDH), and ornithine transaminase (δ-OAT) were evaluated according to Yang, S.L. et al. [30]. To investigate the activity of P5CS, 0.1 g of flower bud samples was homogenized in 1 mL of Tris-HCl buffer, comprising MgCl_2_∙6H_2_O, KCl, EDTA, DTT, and PVP. Subsequently, the homogenized mixture was centrifuged at 8000× *g* for 10 min at 4 °C. Then, 100 µL of the supernatant was combined with an equivalent volume of Tris-HCl buffer containing glutamic acid, ATP, hydroxylamine hydrochloride, and MgCl_2_∙6H_2_O. The reaction was placed at 37 °C for 10 min and then centrifuged at 8000× *g* for 10 min at 25 °C. Finally, 20 µL of the obtained supernatant was mixed with 200 µL of a chromogenic mixture composed of H_2_O, ASA, ammonium molybdate, and H_2_SO_4_. The reaction mixture was left at room temperature for 30 min, and the absorbance was measured at 660 nm. P5CS activity is expressed in µmol/h/g protein. To determine P5CR activity in the flower bud samples, the following procedure was implemented. Initially, 1 mL of Tris-HCl buffer containing NaCl was used to homogenize the flower bud samples, followed by centrifugation at 12,000× *g* for 10 min at 4 °C. Subsequently, 10 µL of the resulting supernatants was combined with 190 µL of a working solution consisting of 90 µL of NAD (nicotinamide adenine dinucleotide) and 90 µL of L-thiazolidine-4-carboxylic acid. The reaction mixture was then incubated at 37 °C in the dark for 30 min. Finally, the absorbance of the reaction was measured at 450 nm, and the enzyme activity is expressed in nmol/min/g protein. To determine δ-OAT activity, 0.1 g of flower bud samples was homogenized in 1 mL of extraction solution (K_2_HPO_4_∙H_2_O-KH_2_PO_4_ buffer solution containing EDTA, mercaptoethanol, and glycerol) and centrifuged at 10,000× *g* for 10 min at 4 °C. Then, 20 µL of supernatant was combined with 60 µL of ornithine, 60 µL of α-ketoglutaric acid, and 60 µL of NADH. The change in absorbance from the start to the end of the reaction was measured at 340 nm and 37 °C. δ-OAT activity is expressed in nmol/min/g protein. To evaluate ProDH activity, 0.1 g of flower bud samples was homogenized in 1 mL of Na_2_HPO_4_-NaH_2_PO_4_ buffer solution with EDTA and centrifuged at 1500× *g* for 15 min at 4 °C. Then, the supernatant was combined with 10 µL of TritonX-100 and centrifuged at 16,000× *g* for 20 min at 4 °C. Finally, 35 µL of supernatant was combined with 15 µL of PMS and 150 µL of a solution consisting of 2.4 mL of Na_2_CO_3_-NaHCO_3_ buffer solution, 0.3 mL of L-proline and 0.3 mL of DCPIP. The decrease in absorbance was monitored at 600 nm at intervals of 10 min; ProDH activity is expressed in U/g protein.

### 2.3. Transcriptome Analysis

Transcriptome analysis was performed according to the method described by Winfield, M.O. et al. [31]. Total RNA was extracted from inflorescence samples by an RNA extraction kit (Tianjin, Beijing, China), and RNA concentration was measured using NanoDrop 2000 (Thermo Fisher Scientific, Wilmington, DE, USA). The integrity of RNA was evaluated using the Agilent Bioanalyzer 2100 system (Agilent Technologies, Santa Clara, CA, USA) and the RNA Nano 6000 detection kit. The total amount of each sample for the starting inventory was 1 μG. According to the instructions provided by the manufacturer, the Hieff NGS Ultima Dual-Mode mRNA Library Prep Kit for Illumina (Yeasen Biotechnology (Shanghai) Co., Ltd., Shanghai, China) was used to generate a sequencing library and add an index to the sequence of each sample. Afterward, the library was sequenced on the Illumina NovaSeq platform to generate 150 bp paired-end sequences. Then, the bioinformatics analysis platform BMKCloud (www.biocloud.net accessed on 15 December, 2022) was used to process the raw data further to obtain valid data (clean data). Next, the Hisat2 tool software (version-2.2.1) was used to analyze and annotate the reference genome. Gene ontology (GO) and KEGG analyses were performed on the DEGs of each sample.

### 2.4. Metabolome Analysis

Metabolome analysis was performed using the following methods described by Tang et al. [32]. For this purpose, 50 mg of flower spike samples was weighted and combined with 1000 μL of an extraction solution. The mixed samples were treated with a 45 Hz grinder and stranded at −20 °C for an hour. Then, they were centrifuged at 4 °C and 12,000× *g* for 15 min, and 500 μL of each supernatant was dried in a vacuum concentrator. At this point, 160 μL of the extraction solution was added to the dried metabolites, and the mixture was vortexed for 30 s. The vortexed samples were subjected to ultrasound in an ice water bath for 10 min and then centrifuged at 4 °C and 12,000× *g* for 15 min. Finally, 120 μL of each supernatant was placed in a 2 mL injection bottle and subjected to machine detection using a Watsch Acquire I-Class PLUS ultra-high-performance liquid chromatographer in tandem with a Watsch Xevo G2-XS QTOF high-resolution mass spectrometer. The detection results were analyzed based on the online METLIN database, a public database, and the self-built database of the Progenesis QI software. (version v2.3) The identified metabolites were annotated using the KEGG and HMDB databases. 

### 2.5. Statistical Analysis

The study used an RCBD (randomized complete block design) with three replications for each biological sample [33]. A two-way ANOVA was conducted, employing Duncan’s multiple range test (DMRT), with a significance level set at *p* ≤ 0.05. This analysis aimed to assess the significance of the data within each group, variations between treatments during the same period, and changes within the same treatment across different time points. This analysis was carried out using SPSS Statistics (version-25.0) software, and Graph Pad Prism (version 8.0.0) was used to prepare all graphical representations. With the R statistics function prcomp (http://www.r-project.org accessed on 20 December, 2022), unsupervised principal component analysis (PCA) was conducted. Before unsupervised PCA, the data were scaled using the unit variance [34]. Heatmaps with dendrograms were generated from the results of HCA (hierarchical cluster analysis). HCA was performed using the ComplexHeatmap R package. A color spectrum represents the normalized signal intensities in HCA [34].

## 3. Results 

### 3.1. Temperature Variation 

**Figure 1:**
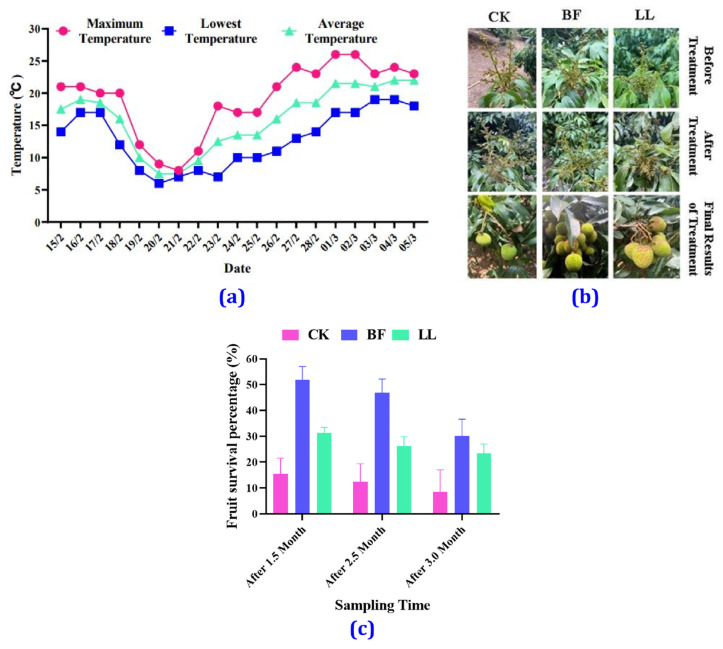
Temperature variance and morphological changes in litchi. (**a**) Temperature records from 15 February 2022 to 5 March 2022 in Yangjiang City, (**b**) developmental stages, and (**c**) fruit survival rate of litchi. Values are expressed as mean ± SD for each treatment.

The temperature variation from 15 February 2022 to 5 March 2022 in Yangjiang City is shown in Figure 1a. On 18 February (1st day), cooling started with a maximum temperature of 20 °C, a minimum temperature of 12 °C, and an average temperature of 16 °C. On 19 February (2nd day), the minimum temperature dropped to 10 °C, whereas on 20 February, the temperature reached 6 °C (lowest point). The temperature began to rise on 22 February and continued to increase daily. In Figure 1a, it can be seen that the temperature decreased rapidly and increased rapidly during this experiment. For three days, the average temperature was below 10 °C. In spring, there were often sudden drops and rises in temperature in Guangdong, which significantly impacted economic crops, especially litchis, during the flowering and fruiting period.

### 3.2. Morphological Changes

In the inflorescence growth stage, the inflorescence length was 20 cm (Figure 1b). After the Bihu (BF) and Liangli (LL) treatments, the inflorescences were yellow-green, with tight clusters of small flowers and healthy growth, while those in the control group (CK) were slightly dark and black, with small flowers drooping and some of the windward flowers shedding. However, the BF group’s small flowers were somewhat wilted, and their color became dark but not black, and the LL group’s inflorescence also appeared wilted and darkened. Still, the color change was not as significant as for the CK group. When the fruit was approaching maturity, it was found that there were only 2–5 (the survival rate was 8.58%) fruits on multiple ears in the CK group, with an average of 4 fruits per ear, a number significantly lower than those observed in the BF group (with an average of 10 fruits per year and a survival rate of 30.08%) and in the LL group (with an average of 7 fruits per year and a survival rate of 23.33%). It can be seen that spraying BF and LL before low temperature occurs can significantly protect the inflorescence and improve fruit setting rate and yield (Figure 1b,c and Table S1).

### 3.3. Biochemical Characterization on Inflorescence Growth 

#### 3.3.1. Antioxidant Enzyme and Malondialdehyde (MDA) Analysis 

**Figure 2:**
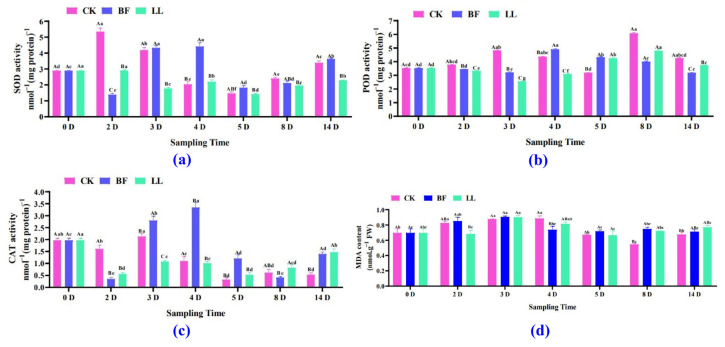
Antioxidant enzyme activity and MDA content in the flower spike. (**a**) SOD activity, (**b**) POD activity, (**c**) CAT activity, and (**d**) MDA content at low temperatures. Values are expressed as mean ± SD for each treatment. Two-way analysis of variance followed by Duncan’s multiple range test; uppercase letters indicate different treatments in the same period, and lowercase letters indicate the same treatment at various times, with *p* at a 0.05 significance level.

SOD activity in the BF group and the LL group was 1.38 and 2.90 nmol^−1^, whereas in the CK group, it was 5.35 nmol^−1^ on the 2nd day of treatment; however, on the 4th day, SOD activity was significantly increased in the BF group compared with the other groups (Figure 2a and Table S2). On the 14th day, there was no significant difference in SOD activity between the CK and BF groups. Still, both groups showed considerably higher SOD activity than the LL group (Figure 2a and Table S2). POD activity reached its highest value in the CK and LL groups (6.08 and 4.80 nmol^−1^) on the 8th day and in the BF group (4.90 nmol^−1^) on the 4th day (Figure 2b and Table S2). But from the 2nd to the 3rd day, POD activity significantly decreased in the BF and LL groups compared with the CK group; then, from the 4th to the 5th day, it increased considerably in the BF group (Figure 2b and Table S2). From the 8th to the 14th days, POD activity significantly decreased in the BF group compared to the CK and LL groups. 

CAT activity varied similarly to SOD and POD activities (Figure 2c and Table S2). The MDA content first tended to increase and then to decrease. On the 2nd, 3rd, and 8th days, it did not significantly increase in the BF group compared to the CK and LL groups. Still, on the 4th day, it significantly decreased in the BF group (Figure 2d and Table S2). On the 8th day, the BF group and the LL group showed an MDA content of 0.75 and 0.72 nmol.g^−1^, and the CK group of 0.55 nmol.g^−1^. On the 14th day, there was no significant difference between the BF group and the CK and the LL groups in MDA content, but the LL group showed a substantial increase in it compared to the CK group (Figure 2d and Table S2). 

#### 3.3.2. Soluble Protein and Proline Content

**Figure 3:**
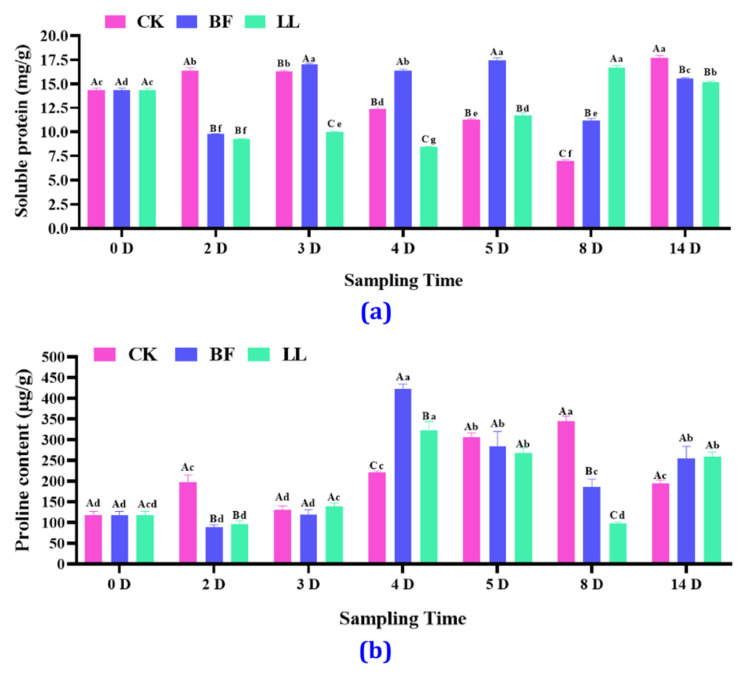
Effects of different PGRs on soluble protein and proline content. (**a**) Soluble protein content, (**b**) soluble proline content. Values are expressed as mean ± SD for each treatment. Two-way analysis of variance followed by Duncan’s multiple range test; uppercase letters indicate different treatments in the same period, and lowercase letters indicate the same treatment at various times, with *p* at a 0.05 significance level.

The soluble protein content in litchi spikes showed an increasing and a decreasing trend. On the 2nd day, it was 9.79 and 9.25 mg/g in the BF and LL groups and 16.34 mg/g in the CK group but on the 3rd day, it was 17.01 mg/g in the BF group and 9.99 mg/g in the LL group (Figure 3a and Table S3). On 4th and 5th days, the soluble protein content in the BF group increased. On the 8th day, it significantly increased by 60.73% and 139.80%, respectively, in the BF and LL groups. On the 14th day, it decreased by 12.13% and 14.42%, respectively, in the BF and the LL groups. The proline content in all treatment groups showed an increasing trend and, in the BF and LL groups, reached the highest values of 422.78 and 321.88 µg/g, whereas in the CK group, it was 221.33 µg/g on the 4th day. There was no significant difference among the three groups on the 3rd, 5th, and 14th days (Figure 3b and Table S3).

#### 3.3.3. Different Soluble Sugars’ Content 

**Figure 4:**
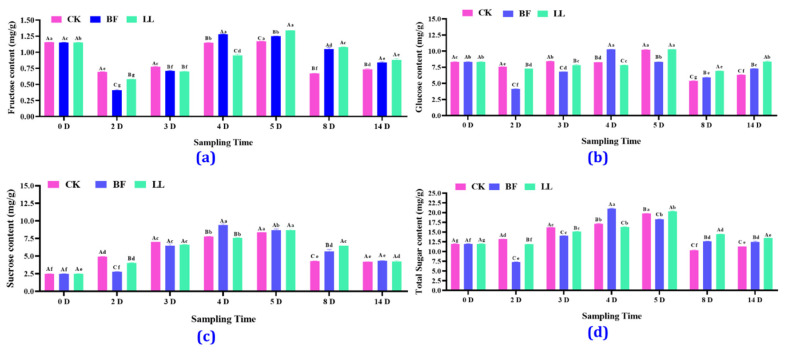
Content of different soluble sugars. (**a**) Soluble fructose content, (**b**) glucose content, (**c**) sucrose content, and (**d**) sugar content in flower spikes at low temperatures. Values are expressed as mean ± SD for each treatment. Two-way analysis of variance followed by Duncan’s multiple range test; uppercase letters indicate different treatments in the same period, and lowercase letters indicate the same treatment at various times, with *p* at a 0.05 significance level.

Regarding the fructose content, the BF group reached the highest content of 1.28 mg/g on the 4th day, while the LL group reached a content of 1.34 mg/g on the 5th day after treatment. On the 2nd and 3rd days, the fructose content decreased in these groups compared to that before treatment, but on the 5th day, it increased in all groups. On the 4th day, the fructose content in the BF group had risen by 12.33% and in the LL group had decreased by 17.10% compared to that in the CK group, while from the 8th to the 14th day (Figure 4a and Table S4), it significantly increased in the BF and LL groups compared to the CK group. For glucose, the results were comparatively similar to those of fructose, whose content tended to decrease first and then to increase (Figure 4b and Table S4); however, the sucrose and sugar content tended to rise first, then drop, and finally stabilize. The BF group had the highest sucrose content of 9.45 mg/g on the 4th day (Figure 4c and Table S4). On the 4th day, the sucrose content in the BF group increased by 21.97% and 24.95%, compared with that in the CK and the LL groups. Regarding the sugar content, the BF group showed the highest increase of 22.52%, while the LL group showed a decrease of 4.89% compared with the CK group on the 4th day (Figure 4d and Table S4). 

#### 3.3.4. Sucrose Synthetase and Sucrose Phosphate Synthetase Analysis

**Figure 5:**
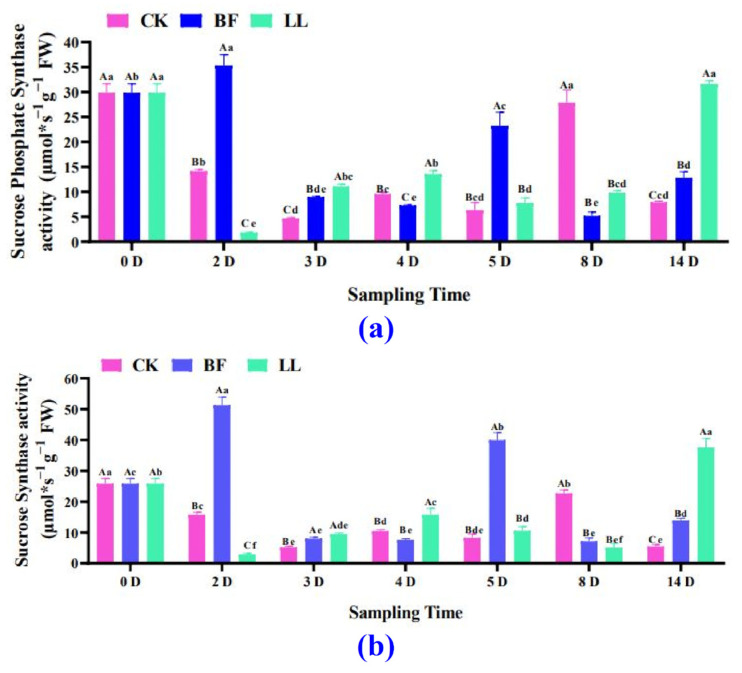
Variations in sucrose and sucrose phosphate synthetase activities. (**a**) Soluble phosphate synthase activity, (**b**) soluble sucrose synthetase activity in flower spikes at low temperatures. Values are expressed as mean ± SD for each treatment. Two-way analysis of variance followed by Duncan’s multiple range test; uppercase letters indicate different treatments in the same period, and lowercase letters indicate the same treatment at various times, with *p* at a 0.05 significance level.

Both sucrose synthetase and sucrose phosphate synthase activity decreased and then increased in the CK and LL groups. In contrast, in the BF group, they showed an overall fluctuating trend, rising, falling, and then rising again (Figure 5a and Table S5). In Figure 5a, it can be seen that the overall trend of sucrose phosphate synthase activity is similar to that of sucrose synthase activity (Figure 5b). 

#### 3.3.5. Key Proline-Related Enzyme Activities

**Figure 6:**
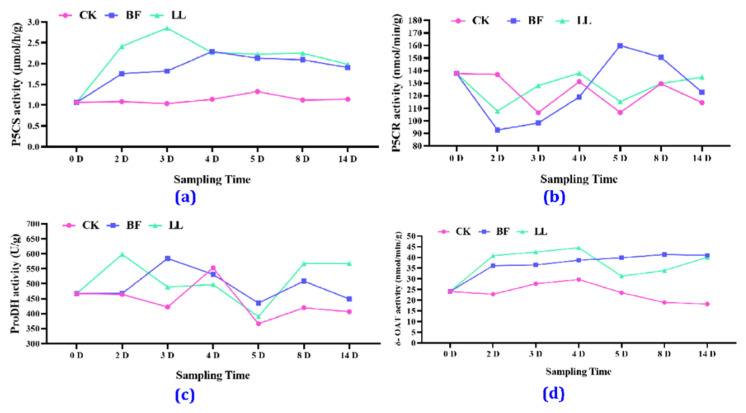
Effects of different PGRs on key proline-related enzyme activities. (**a**) P5CS activity, (**b**) P5CR activity, (**c**) ProDH activity, and (**d**) δ-OAT activity in flower spikes at low temperatures.

In Figure 6a and Table S6, it can be seen that there was no significant change in the activity of P5CS in the CK group during the experiments. Still, the BF and the LL treatment groups showed an increase, a decrease, and then a gradual stabilization of P5CS activity, and the BF group showed its highest P5CS activity on the 4th day. In Figure 6b and Table S6, it can be seen that P5CR activity in the BF group reached its highest on the 5th day, whereas ProDH activity reached its highest on the 3rd day (Figure 6c and Table S6). In Figure 6d and Table S6, it can be seen that δ-OAT activity showed an upward trend and then gradually stabilized. 

### 3.4. Transcriptome Analysis 

#### 3.4.1. Transcriptome Sequencing Data Analysis 

**Figure 7:**
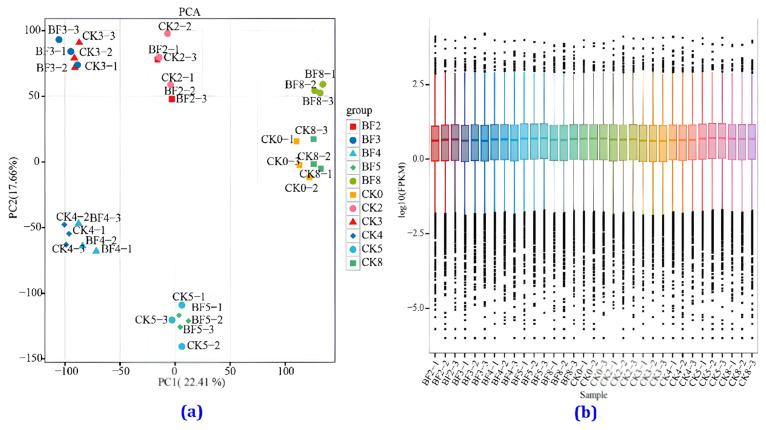
Principal component analysis (PCA) and gene expression level distribution in the transcriptome. (**a**) PCA between the CK and the BF groups, (**b**) gene expression in flower spikes under low-temperature conditions treated with PGRs.

Each sample’s eukaryotic reference transcriptome (RNA sequence) analysis yielded clean data, with all samples reaching 5.70 GB. Sequence alignment was performed with the reference genome, with alignment efficiency ranging from 89.79% to 91.43%. The base percentage of Q30 was 93.00% or higher. The proportion of bases with a clean data quality value greater than or equal to 30 was above 92%. Figure 7 shows that under low-temperature conditions, there was a significant difference between the samples in different sampling days.

Conversely, on the same day, the differences between the CK and BF groups were relatively small. The PCA graph in Figure 7a shows that the samples in each study period were well separated, and there were no significant outliers in the internal samples in each period. The use of transcriptome data to detect gene expression in samples has high sensitivity. The box plot of the gene expression levels in each sample shows the dispersion of the gene expression level distribution in individual samples and allows for comparing the overall gene expression levels in different samples (Figure 7b).

#### 3.4.2. Differentially Expressed Genes (DEGs) and Gene Ontology (GO) 

Differentially expressed genes (DEGs) were screened in the CK (CK0, CK2, CK3, CK4, CK5) and BF (BF2, BF3, BF4, BF5) groups based on the *p* value. The CK groups presented more downregulated than upregulated DEGs (Figure S1a–e). The comparison of the CK0 vs. CK2, CK0 vs. CK3, CK0 vs. CK4, and CK0 vs. CK5 groups identified 5025, 8531, 8660, and 5427 DEGs, respectively, with 1340 overlapping DEGs (Figure S1), whereas the comparison of the BF0 vs. BF2, BF0 vs. BF3, BF0 vs. BF3, BF0 vs. BF4 groups identified 3774, 8135, 7321, and 5403 DEGs, respectively, with 1243 overlapping DEGs (Figure S2). There were more downregulated DEGs than upregulated DEGs (Figures S1e and S2e). These differentially expressed genes are chill-responsive genes (CRGs). The CRGs were annotated using the GO (gene ontology) database and classified into three categories: biological processes, molecular functions, and cellular components (Figure S3a,b) related to litchi growth and development. The proportion of DEGs annotated to molecular functions in the different control and treatment groups was relatively small; a higher number of genes were annotated to binding and catalytic activity functions.

#### 3.4.3. KEGG Analysis

KEGG (Kyoto Encyclopedia of Genes and Genomes) is a database that systematically analyzes gene function and genomic information. KEGG pathway analysis showed that the CK group was significantly enriched in CRGs involved in phenylpropanoid biosynthesis, plant circadian rhythm, DNA replication, homologous recombination, and mismatch repair pathways (Figure S4a). Regarding the BF group, the differentially expressed genes (DEGs) related to the PGR response were mostly involved in the DNA replication route, plant hormone signal transduction pathway, and plant circadian rhythm pathway (Figure S4b).

#### 3.4.4. Heat Map Analysis

Regarding plant hormone signal transduction, 39 CK genes and 43 BFgenes were related to these signal transduction pathways (Figures S5a,b and S6a–c). Among the 39 genes, 9 were related to brassinosteroid (BR) signal transduction, including 3 CYCD3 (*Arabidopsis* cyclin D3), 2 TCH4 (*Arabidopsis* TCH [for touch]), and 4 BRI1 (brassinosteroid-insensitive 1) genes, 6 were associated with auxin signal transduction (indoleacetic acid [IAA]; 1 GH3 [Gretchen Hagen 3], 2 SAUR [small auxin-up RNA], 2 AUX/IAA [auxin/IAA], and 1 ARF [auxin response factors] genes), 3 were linked to cytokinin signal transduction (CTK; 1 CRE1 [cytokinin response 1] and 2 ARR-B [type B *Arabidopsis* response regulators] genes), 6 were related to gibberellin (GA) signal transduction (4 DELLA [(aspartic acid, glutamic acid, leucine, leucine, and alanine], 1 TF, and 1 GID1 [gibberellin-insensitive dwarf1] genes), 4 were related to jasmonic acid signaling (JA) (3 MYC2 [myelocytomatosis], 1 JAZ [jasmonate-ZIM domain] genes), 1 was associated with salicylic acid (SA) signaling (TGA [TGACG-binding] gene), 7 were linked to abscisic acid signal transduction (ABA) (5 PP2C [type 2C protein phosphatases] and 2 ABF [ABRE-binding factor] genes), and 3 were linked to ethylene signal transduction (ETH) (1 CTR1 [constitutive triple response 1], 1 SIMKK [salt stress-inducible MAPK kinase], and 1 ETR [ethylene receptor] genes). Among the 43 genes, 9 were associated with BR, 8 with IAA, 4 with CTK, 6 with GA, 4 with JA, 2 with SA, 6 with ABA, and 4 with ETH signaling, respectively (Figure S6). 

In the starch and sucrose metabolism pathway, the CK group was enriched for 25 PGR response genes, including glycogen phosphorylase (GP, EC: 2.4.1.1), β-fructofuran glycosidase (fruct2, EC: 3.2.1.26), β-glucosidase (BGL, EC: 3.2.1.21), α-amylase (AMY, EC: 2.4.1.13), trehalose 6-phosphate synthase (TPS, EC: 2.4.1.15), and sucrose synthase (SUS, EC: 2.4.1.13) genes (Figure S6), whereas the BF group was enriched for 23 PGR response genes including phenylpropanoid biosynthesis (ko00940) and circadian rhythm genes (Figure S6). 

#### 3.4.5. Comparison of the Transcriptome between the CK and the BF Groups

**Figure 8:**
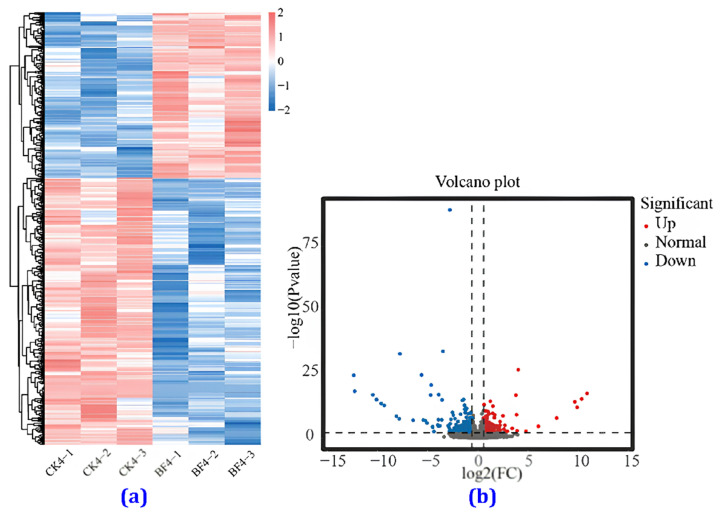
**(a**) Heat map of gene expression and volcano plot of differentially expressed genes. (**a**) Heat map of gene expression, (**b**) volcano plot of differentially expressed genes in flower spikes in the CK and BF groups four days after treatment.

The most significant difference was observed on the 4th day of treatment between the BF and the CK groups compared to other time points. Therefore, the CK4 and the BF4 groups on the 4th day were used as samples for differential transcriptome analysis. In the hierarchical clustering heat map (Figure 8a), it can be seen that under low-temperature conditions, there was a significant difference in the gene expression patterns between the BF group sprayed with the Bihu PGR and the CK group sprayed with water, and more downregulated genes than upregulated ones were observed. There were 198 upregulated genes and 319 downregulated genes (Figure 8b).

**Figure 9:**
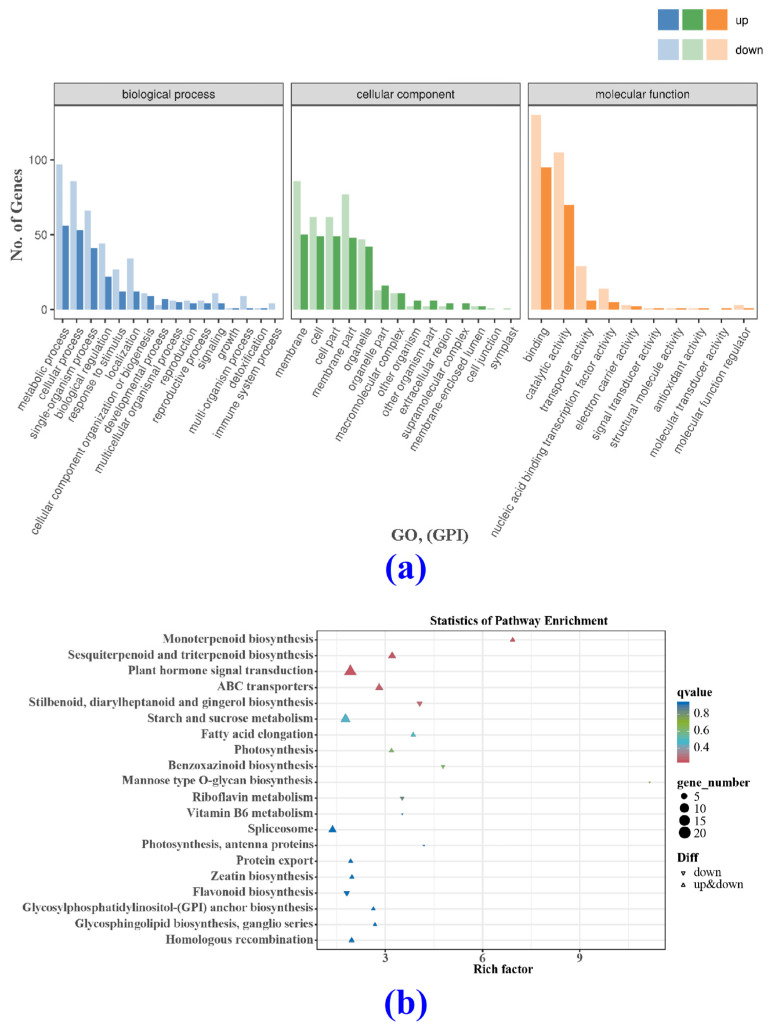
GO classification map and KEGG bubble plots analysis. (**a**) GO classification map, (**b**) KEGG bubble plots of differentially expressed genes in flower spikes in the CK and BF groups 4 days after treatment.

The differentially expressed genes identified in the CK4 and BF4 groups were analyzed using the GO database. It was found that the majority of differentially expressed genes were associated with the biological process and cell composition categories. In contrast, fewer were found in the molecular function category (Figure 9a). By analyzing the differentially expressed genes mentioned above through the KEGG database (Figure 9b), it was found that genes associated with the biosynthesis of monoterpene compounds (ko00902), sesquiterpenoid and triterpenoid biosynthesis (ko00909), plant hormone signal transduction (ko04075), ABC transporters (ko02010), and the biosynthesis of stilbenoids, diarylheptanoids, and ginger alcohols (ko00945) were prominent. In addition to these pathways, starch and sucrose metabolism (ko00500) and spliceosome (ko03040) pathways also showed gene enrichment.

**Figure 10:**
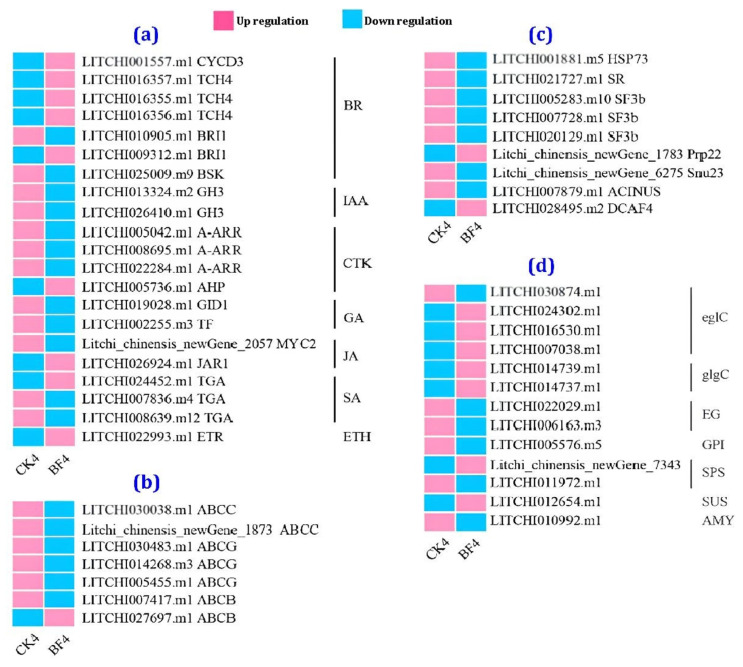
Heat map of the differentially expressed genes in the CK and BF groups on the 4th d after treatment. (**a**) Heat map comparison of enriched genes related to plant hormone signaling pathways, (**b**) heat map comparison of enriched genes related to ABC transporters, (**c**) heat map comparison of enriched genes related to the spliceosome, (**d**) heat map comparison of enriched genes related to starch and sucrose metabolism.

Subsequently, 21 genes related to the plant hormone signaling pathway, 7 genes associated with ABC transporters, 9 genes related to the spliceosome, and 13 genes related to starch and sucrose metabolism were selected from the differentially expressed genes in the CK4 and BF4 groups. The 21 genes related to plant hormone signaling, identified as the candidate genes induced by PGRs (Figure 10a), included 7 BR-, 2 IAA-, 4 CTK-, 2 GA-, 2 JA-, 3 SA-, and 1 ETH-related genes. The differentially expressed genes related to BR and ETH signal transduction were mostly upregulated, while those related to other hormone signaling pathways were downregulated. Among the candidate genes mediating the PGR responses, seven ABC transporter-related genes and nine splice-related genes were identified, mostly showing downregulation (Figure 10b,c). In contrast, the 13 identified genes related to starch and sucrose metabolism were up- and downregulated (Figure 10d).

### 3.5. Metabolome Analysis 

#### 3.5.1. Differentially Accumulated Metabolite (DAM) Analysis

The metabolite profile of flower spikes was analyzed using an UPLC-ESI-MS/MS system to understand the differences in metabolite levels between the BF and CK groups. The DAMs in the CK0 vs. the CK4 group were 1469, whereas in the CK0 vs. the CK3 group they were 1008, in the CK0 vs. the CK2 group they were 1057, in the CK0 vs. the CK5 group they were 1129 (Figures S8 and S9). Among the four CK groups, there were 408 overlapping DAMs, defined as low-temperature-responsive metabolites. The first 20 metabolic pathways in the HMDB database related to DAMs involved in the low-temperature response of litchi flower spikes are shown in Figure S8. Regarding the BF group, the DAMs in the BF0 vs. the BF4 group were 1253, whereas those in the BF0 vs. the BF5 group were 1363; the comparison between the BF0 and the BF2 groups revealed the smallest number of DAMs, corresponding to 1027, and that between the BF0 and the BF3 groups showed 1112 DAMs. Among the four BF groups, 422 overlapping DAMs were defined as PGR-responsive metabolites. The top 20 metabolic pathways annotated in the HMDB database related to DAMs involved in the growth regulator response of litchi flower spikes are shown in Figure S7a,b.

#### 3.5.2. KEGG Analysis

In the KEGG analysis of the BF groups, it was observed that there were 38 metabolites with significant differences annotated in the KEGG database, distributed in 20 metabolic pathways (Figures S10 and S11). Among them, 26.3% of the DAMs participated in the biosynthesis of other plant secondary metabolites. We found that 23.7% of the DAMs were involved in lipid metabolism, and most DAMs in this pathway were significantly enriched. In addition, 18.4% of the DAMs were involved in amino acid metabolism, and the most abundant DAMs were found in the tryptophan metabolism pathway. However, in the BF groups, it was seen that among the top 20 metabolic pathways annotated in the KEGG database, lipid metabolism accounted for the highest proportion, with 30 metabolites enriched with significant differences (Figure S11). Secondly, 18 differential metabolites were involved in organic oxygen compound-related pathways, and 17 were involved in pathways associated with propanol lipids, including benzene and its substituted derivatives, flavonoids, carboxylic acids and their derivatives, etc. Among them, DAMs linked to the metabolism of alanine (beta-alanine metabolism), the biosynthesis of phenylpropanoid (phenylpropanoid biosynthesis), linoleic acid metabolism, and histidine metabolism were the most significantly enriched, followed by DAMS involved in the biosynthesis of various alkaloids, the degradation of limonene and pinene, α-linolenic acid metabolism, flavone and flavonol biosynthesis, and purine metabolism.

#### 3.5.3. Comparison of the Metabolome between the CK and BF Groups

**Figure 11:**
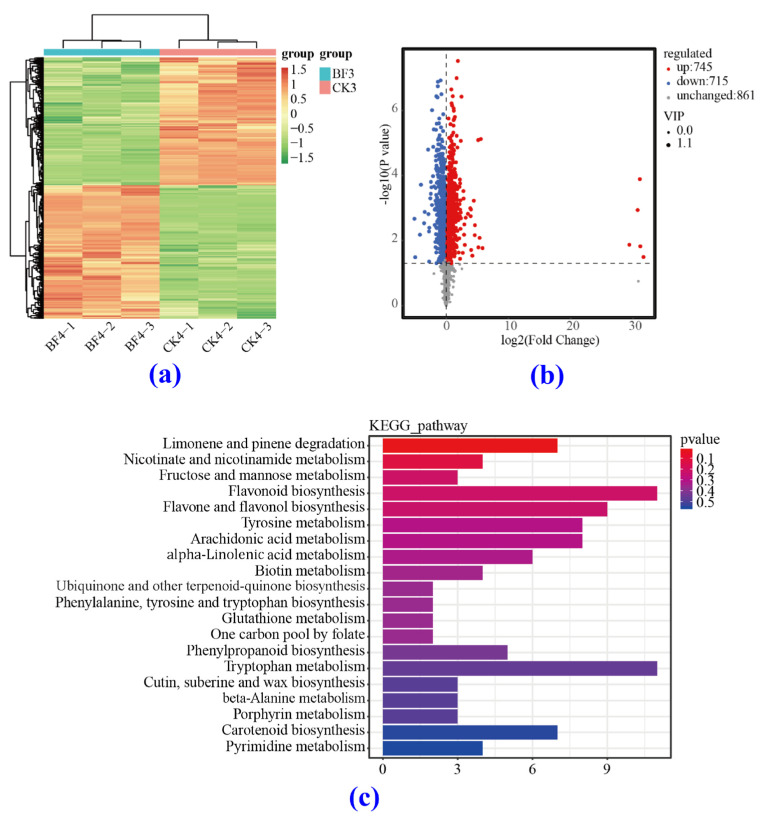
Heat map and differentially accumulated metabolite analysis between the CK and the BF groups 4 d after treatment. (**a**) Heat map analysis, (**b**) DAM regulation, and (**c**) DAM KEGG classification plots.

The hierarchical clustering heat map (Figure 11a) shows that under low-temperature conditions, there was a significant difference in metabolite content between the BF and the CK groups on the 4th day after treatment. There were 745 differentially accumulated metabolites with increased content and 715 differentially accumulated metabolites with decreased content (Figure 11b). Overall, there were more DAMs with increased content than DAMs with decreased content.

By analyzing the above differentially accumulated metabolites through the KEGG database (Figure 11c), it was found that they were enriched in metabolites involved in the degradation of limonene and pinene (ko00903), in the metabolism of nicotinic acid and nicotinamide (ko00760), and in the metabolism of fructose and mannose (ko00051). The biosynthesis of flavonoids (ko00941) and the biosynthesis of flavonoids and flavonols (ko00944) were the most prominent pathways. In addition to these pathways, there was also significant enrichment of DAMs involved in tyrosine metabolism (ko00350), tryptophan metabolism (ko00380), and carotenoid biosynthesis (ko00906). 

### 3.6. Correlation Analysis between Transcription and Metabolism for the CK and BF Groups

**Figure 12:**
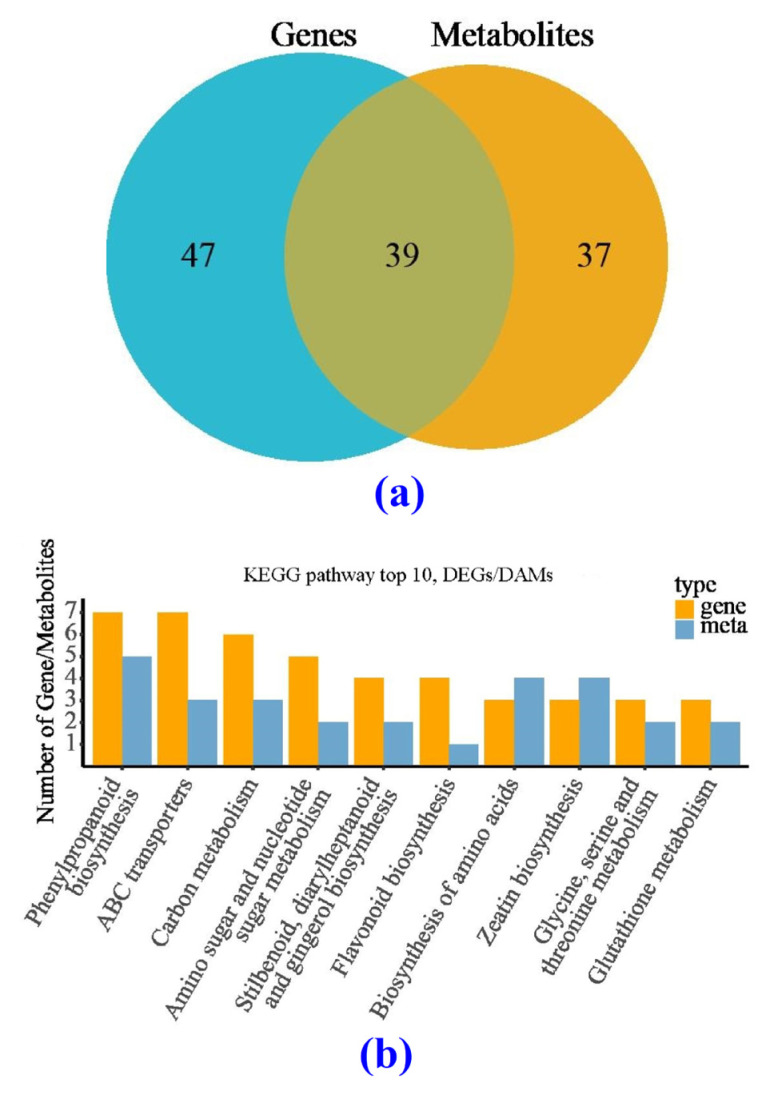
The common metabolic enrichment pathways from DEG and DAM analysis. (**a**) Volcano plot analysis, (**b**) the top ten metabolic pathways.

After KEGG enrichment, a total 39 common enrichment pathways were obtained for DAMs and DEGs (Figure 12a). The top ten metabolic pathways with high reliability were phenylpropanoid biosynthesis, ABC transporter protein-related pathways, carbon metabolism, amino sugar and nucleotide sugar metabolism, styrene, diarylheptane and gingerol biosynthesis, flavonoid biosynthesis, amino acid biosynthesis, zeatin biosynthesis, glycine, serine, and threonine metabolism, and glutathione metabolism (Figure 12b). These pathways are mostly related to plant response to stress.

**Figure 13:**
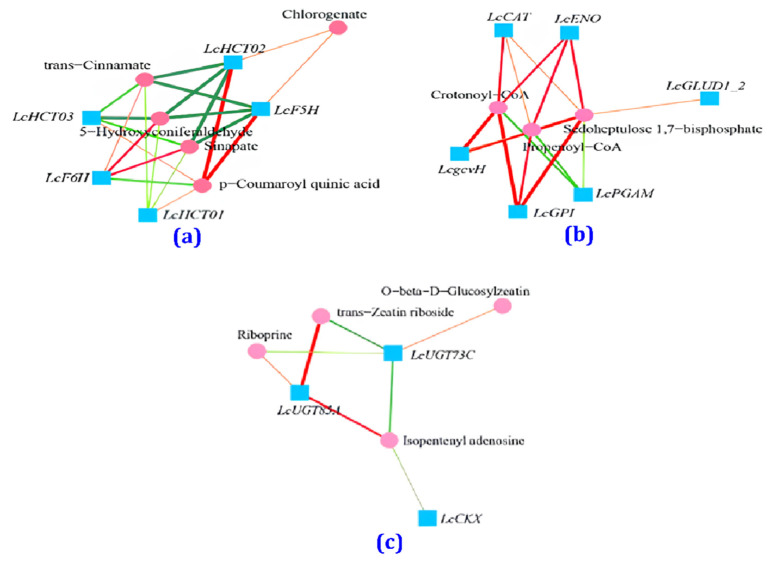
Correlation network diagram between transcription and metabolism for the CK and BF groups. (**a**) Phenylpropyl biosynthesis pathway network, (**b**) carbon metabolism pathway network, (**c**) zeatin biosynthesis pathway network.

In the phenylpropyl biosynthesis pathway, seven DEGs encoded four enzymes: HCT (shikimate O-hydroxycinnamoyltransferase) and F5H (ferulate-5 hydroxylase) were upregulated, whereas POD (peroxidase) and F6H (feruloyl CoA 6-hydroxylase) were downregulated by low temperature (Figure S12a,b). At the same time, a total of five DAMs were detected, among which chlorogene and p-coumaroyl quinic acid showed increased content, while 5-hydroxyconiferaldehide, sinapate, and trans-cinnamamate showed decreased content under low temperature (Figure S12c). The correlation between DEGs and DAMs was above 0.8 (Figure 13a). In the case of the carbon metabolism pathway, it can be seen that six DEGs (ENO, CAT, gcvH, GLUD1_2, PGAM, and GPI) encode enzymes. Among them, LcCAT may be a negative regulator in the process of CAT enzyme production. At the same time, three DAMs (crotonoyl-CoA, propenoyl-CoA, and sedhoptulose 1,7-bisphosphate) related to this pathway were detected (Figure S13c), and their contents were all reduced. The correlation between DEGs and DAMs showed that LcENO, LcCAT, LcgcvH, and LcGPI positively regulate these three metabolites, while LcGLUD1_2 positively regulates only sedoheptulose 1,7-bisphosphate, and LcPGAM negatively regulates the three metabolites (Figure 13b). Considering the zeatin biosynthesis pathway, three enriched DEGs encode three related enzymes: CKX (cytokine dehydrogenase, K00279), UGT85A (UDP glucose transfer 85A, K23452), and UGT73C (UDP glucose transfer 73C, K13496). Except for LcUGT85A, which was downregulated under low-temperature stress, the other two genes were upregulated. After spraying BF, LcUGT85A was upregulated, while LcCKX and LcUGT73C were downregulated. At the same time, four DAMs were detected in this pathway (Figure S14b). Under low-temperature stress, the contents of trans-zeatin riboside diphosphate and O-beta-D-glucosyl-trans-zeatin increased, while the contents of isopentenyl adenosine and riboprine decreased. After spraying Bihu, the opposite changes in the levels of these metabolites were observed. The correlation between DEGs and DAMs, showed that LcUGT73C positively regulates O-beta-D-glucosyl-trans-zeatin and negatively regulates trans-zeatin riboside diphosphate, riboprine, and isopentenyl adenosine (Figure 13c). On the contrary, LcUGT85A positively regulates trans zeatin riboside diphosphate, riboprine, and isopentenyl adenosine. At the same time, LcCKX positively regulates isopentenyl adenosine.

## 4. Discussion

**Figure 14:**
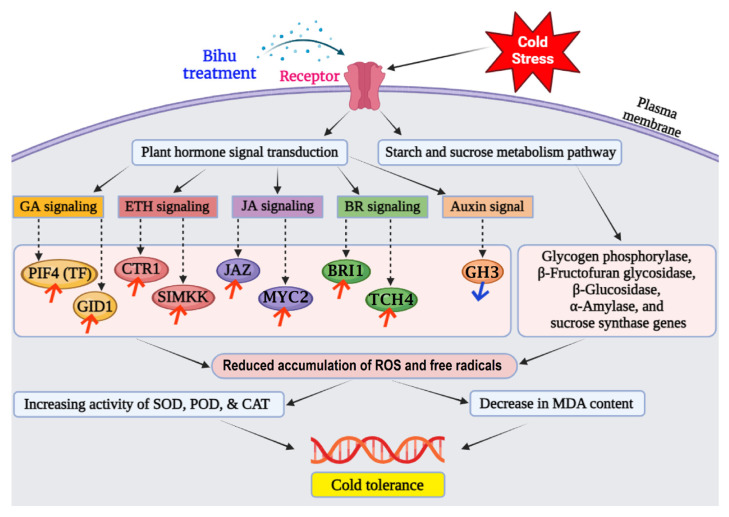
A proposed model describing the signaling pathways involved in cold tolerance in litchi flower after spraying Bihu. Here, the red arrow indicates a significant expression or upregulation of the respective gene, and the blue arrow indicates the downregulation of the corresponding gene.

The global climate change is greatly impacting the production of cultivated crops and fruits. Low-temperature stress affects fruits’ nutritional and reproductive growth, decreasing their yield and quality. Although many scientists believe that artificial gene transfer can be used to solve abiotic problems, this methodology raises many concerns. On the other hand, the Bihu treatment can spontaneously regulate flower inflorescence development genes under low temperatures. Usually, under low-temperature stress, plants may eliminate reactive oxygen species (ROS) and establish tolerance by effectively increasing the antioxidant activity of SOD, POD, and CAT (Figure 14). ROS production also causes intracellular lipid peroxidation, leading to the production of MDA.

In our present study, the Bihu treatment was more effective than Liangli and clean water in terms of fruit production, measured by flower inflorescence growth and number of fruits developed. After Bihu and Liangli spraying, the observed changes in soluble sugar, protein, and proline content positively correlated with plant stress resistance [35]. In this study, we found that SOD, POD, and CAT production increased in flower spikes by varying degrees from the 2nd to the 5th day at low temperatures, which reduced damage and increased the soluble matter and proline content. Similar results were reported in [36]. Researchers also suggested that the induction of the DREB1 gene leads to the expression of many cold-induced genes [37,38]. In Arabidopsis, the PIF3, PIF4, and PIF7 proteins inhibited the expression of DREB1 [39,40,41]. Due to the adverse effects of the overexpression of the DREB1 gene on plant growth, DREB1 inhibitors play an important role in controlling the balance between plant growth and cold tolerance. In our study, we also identified the PIF4 (TF) protein in the GA signaling pathway that may initiate flowering under cold stress (Figure 14). The downregulation of the DELLA protein upstream of the TF protein could be due to the high expression of GA and receptor binding to form the GA-GID1 complex, causing DELLA cleavage [42]. In contrast, the upregulation of GID1 expression increases GA sensitivity [43]. 

At the same time, it can be observed that ETH signaling-related genes such as CTR1 and SIMKK were upregulated in litchi inflorescence under low-temperature stress, and the stress response JA signaling-related genes JAZ and MYC2 were also induced. As a key pathway for regulating the response to low-temperature stress, the BR signaling pathway was significantly activated, with the upregulation of the expression of its associated BRI1 protein in litchi flower spikes under low-temperature stress (Figure 14). This could be because the activated BRI1/BAK1 complex can phosphorylate and activate G-proteins, thereby regulating the soluble sugar content [44]. In this study, the upregulation of genes related to increased gibberellin, brassinolide, and abscisic acid content indicated that this mechanism can promote litchi cold tolerance under low-temperature stress. At the same time, low-temperature stress was also found to induce the expression of plant hormone-related proteins such as auxin, cytokinin, salicylic acid, jasmonic acid, and ethylene, causing changes in endogenous plant hormone content and affecting the growth and development of litchi inflorescence. Starch and sucrose metabolism are important response pathways in low-temperature stress. In this study, low-temperature-responsive genes, including glycogen phosphorylase, β-fructofuran glycosidase, β-glucosidase, α-amylase, and sucrose synthase genes, were differentially expressed under low-temperature stress (Figure 14).

In addition, in this study, low-temperature-responsive genes were mostly enriched in pathways related to secondary metabolites, lipid metabolism, and amino acid metabolism, especially tryptophan metabolism, whose differentially accumulated metabolites were the most abundant. It was reported that many plant stress-responsive metabolites are synthesized through amino acid metabolism pathways [45], and the tryptophan metabolism pathway is an important component of the plant immune system [46]. Researchers found that tryptophan synthase subunit 1 (TSB1) participates in the coordination of Trp and ABA, thereby affecting plant growth and abiotic stress responses. In addition, we found that TSB1 is associated with and inhibits β-glucosidase 1 (BG1), which hydrolyzes glucose-coupled ABA into active ABA. In the amiR-TSB1 line, mutation of BG1 reduced ABA accumulation and enhanced stress tolerance. In summary, we found that TSB1 plays a key coordinating role in plant growth and the stress response by balancing the steady-state level of Trp and ABA [47].

Bihu is a PGR composed of gibberellin (GA), indoleacetic acid (IAA), and brassinolide (BR), which can enhance various types of plant stress resistance. According to reports, Bihu can increase the accumulation of dry matter in the young roots and seedlings of rice, promote the development of strong roots and seedlings, and improve their low-temperature resistance. Research showed that GAs can encourage cell elongation and division and are synthesized from a trans-geranylgeranyl compound. In GA signal transduction, the activity of the DELLA protein is the core [48]. GA metabolism [49] and signal transduction [50] are both targets of cold stress, and there is evidence that CBF is also involved. In Arabidopsis, tobacco, and tomato, excessive expression of CBFs leads to a decrease in bioactive GA, which is related to growth inhibition and late flowering, and whose level can be restored by applying exogenous GA [51,52,53]. Other studies provided evidence that GA signaling components can affect plant responses to low-temperature stress, as in Arabidopsis [49,50] and rice [54], GA-sensitive and GA-dependent mutants can alter cold tolerance. Because PIF4 can also control the biosynthesis of coenzymes under high temperatures [55] and its activity is regulated by brass steroids (BRs) [56], the second type of growth-promoting hormones, PIF4, and excess factors may serve as central nodes for integrating multiple environmental stimuli into the growth process. In this study, during the same period of low-temperature stress, there was significant expression of GID1 and TF in litchi flower heads after spraying the Bihu regulator and water (Figure 14). 

According to reports, BRs are a class of hormones that closely interact with GAs and have a clear role in promoting plant growth [57]. No evidence suggests a synergistic effect between BRs and GAs in low-temperature stress. However, in contrast to GAs, BRs are believed to actively control cold stress responses, as some scholars demonstrated that applying BR can improve the cold tolerance of many plants, including cold-sensitive crops such as corn and cucumber [58,59,60]. In addition, using BR to treat Arabidopsis at low temperatures can enhance CBF1 and the CBF target COR47 expression, indicating that BR can promote CBF expression and cold tolerance [61]. In this study, the expression of the BRI1 gene was upregulated and downregulated, while the TCH4 (XET) gene was upregulated. 

Studies showed that a large number of auxin regulatory genes are affected by cold in Arabidopsis and rice [62,63]. For example, auxin analogs under low-temperature stress can induce the accumulation of cold-resistant metabolites and soluble sugar in rapeseed [64]. Research reports showed decreased IAA content after low-temperature treatment [65]. Still, some studies found that the IAA content increased after one day of low-temperature treatment and remained elevated for five days [66]. This treatment was related to an increase in the expression of the YUCCA family auxin biosynthesis genes and a decrease in the expression of the OsGH3 family genes, which catalyze the inactivation of rice auxin [66]. In this study, the expression of the GH3 gene was downregulated in litchi inflorescence after spraying the Bihu regulator (Figure 14), which is similar to the results in rice, indicating that the auxin levels can be increased by activating auxin biosynthesis and inhibiting auxin inactivation.

Low-temperature stress can lead to a large accumulation of reactive oxygen species (ROS) in plants, breaking the original dynamic balance and leading to the oxidation of biofilms, proteins, and nucleic acids, damage to plant tissues, or cell death [67,68]. Plants can directly remove excess ROS through enzymatic and non-enzymatic antioxidant defense systems [69,70] as well as by regulating plant metabolism, such as plant hormone signal transduction [71], glutathione metabolism [72], and β-alanine metabolism [73]. In this study, differentially accumulated metabolites were mostly enriched in plant hormone signal transduction and β-alanine metabolism. Some scholars reported that exogenous 5-aminolevulinic acid (ALA) can activate glutathione metabolism in tomato seedling roots under low-temperature stress. It can activate glutathione metabolism and β-alanine metabolism, further enhancing the ability of tomato seedling roots to scavenge ROS and improve the low-temperature tolerance of tomato seedlings [74]. So, spraying the Bihu growth regulator before the onset of low temperatures can activate β-alanine metabolism and enhance the tolerance of litchi flower heads to low temperatures.

According to the experimental results, it can be seen that spraying Bihu before cooling onset can increase the antioxidant enzyme activity in litchi flower spikes, thereby reducing the accumulation of reactive oxygen species and free radicals and the related damage to cell membranes, promoting cell repair, helping the spikes adapt to low-temperature environments, and reducing the damage of low-temperature stress to them. In addition, after 4 days, the MDA content in the BF group was significantly lower than that in the CK group, which could be due to the increased antioxidant enzyme activity in litchi flower spikes after spraying Bihu, reducing the production of ROS and leading to a decrease in MDA content. This phenomenon indicates that spraying Bihu before cooling occurrence can improve the cold resistance of litchi flower heads (Figure 14). Moreover, during the low-temperature period, the soluble protein content in the BF group was higher than that in the LL and CK groups. Therefore, spraying Bihu before low-temperature onset had a better protective effect on litchi flower heads than spraying Liangli.

In summary, during the low-temperature period, the proline content of the three groups of litchi flower heads increased, indicating that litchi flower heads can increase their intracellular solute concentration by increasing the proline content in their body, thereby reducing the freezing point of cells, avoiding their excessive dehydration, and reducing the harm of low temperature to the body. Spraying Bihu and Liangli can significantly increase the proline content in litchi flower spikes. But, according to this study, spraying Bihu has a better effect than spraying Liangli.

## 5. Conclusions

Litchi inflorescence growth and fruit production are enhanced by Bihu and Liangli treatments during resistance to low-temperature stress with the help of enhanced protein, sugar, proline, and MDA content in the flower heads and of increased P5CS, which is involved in proline metabolism, and δ-OAT enzymatic activities. These treatments can support inflorescence resistance to cold stress by activating the SOD, POD, and CAT enzymes and inducing the expression of cold-response genes. Litchi inflorescence development was significantly increased after spraying Bihu. Metabolites were also regulated by the Bihu treatment. The genes that conferred cold resistance after the treatments were also identified, and a hypothesis was presented. These results provide valuable information for understanding the molecular mechanism of Bihu treatment-mediated low-temperature stress resistance during flower development. This study also offers a strategy to modulate plant growth using Bihu to produce litchi. 

## Data Availability

The datasets are available in the Sequence Read Archive (SRA) database of NCBI with the number PRJNA1035920 (https://www.ncbi.nlm.nih.gov/search/all/?term=PRJNA1035920 accessed on 5 November, 2023). All of the data have been released.

## Supplementary Materials

The following supporting information can be downloaded at: https://www.mdpi.com/article/10.3390/metabo14040223/s1, Figure S1: Differential gene expression distribution in flower spikes in control groups under low-temperature conditions; Figure S2: Differential gene expression distribution in flower spikes under low temperature conditions in Bihu treatment groups; Figure S3: GO classification map of low-temperature response genes in flower spikes; Figure S4: KEGG classification map of low-temperature response genes in flower spikes; Figure S5: Heat map of plant growth regulator candidate gene enrichment pathways in CK groups under low temperature; Figure S6: Heat map of plant growth regulator candidate gene enrichment pathways in BF groups under low temperature; Figure S7: Top 20 classification chart of metabolites in flower spikes under low-temperature conditions; Figure S8: Differential Metabolite content distribution of flower spikes under low temperature conditions; Figure S9: Differential metabolites of flower spikes under low temperature conditions treated with plant growth regulators; Figure S10: Metabolite content distribution of flower spikes under low temperature conditions; Figure S11: KEGG bubble plots of metabolites in flower spikes under low temperature conditions; Figure S12: Correlation analysis of thermal map of DEGs and DAMs for phenylpropyl biosynthesis pathway; Figure S13: Correlation analysis of the thermal map of DEGs and DAMs for Carbon metabolism pathway, and Figure S14: Correlation analysis of thermal map of DEGs and DAMs for Zeatin biosynthesis pathway; Table S1: Fruit Survival Percentage (%); Table S2: SOD, POD, CAT activity, and MDA content; Table S3: Soluble Protein and Proline content; Table S4: Fructose, glucose, sucrose, and total sugar content; Table S5: Sucrose Phosphate Synthase activity and Sucrose Synthase activity, and Table S6: P5CS, P5CR, ProDH, and δ- OAT activity.
