# Supplementary material for: Cryoprotectant-Mediated Cold Stress Mitigation in Litchi Flower Development: Transcriptomic and Metabolomic Perspectives
Source: Metabolites. 2024 Apr 15;14(4):223. doi: 10.3390/metabo14040223 (PMC11052034; doi:10.3390/metabo14040223)
Supplement: Supplementary file 1 [file metabolites-14-00223-s001.zip › metabolites-2920917-supplementary.pdf]

Supplementary Figures

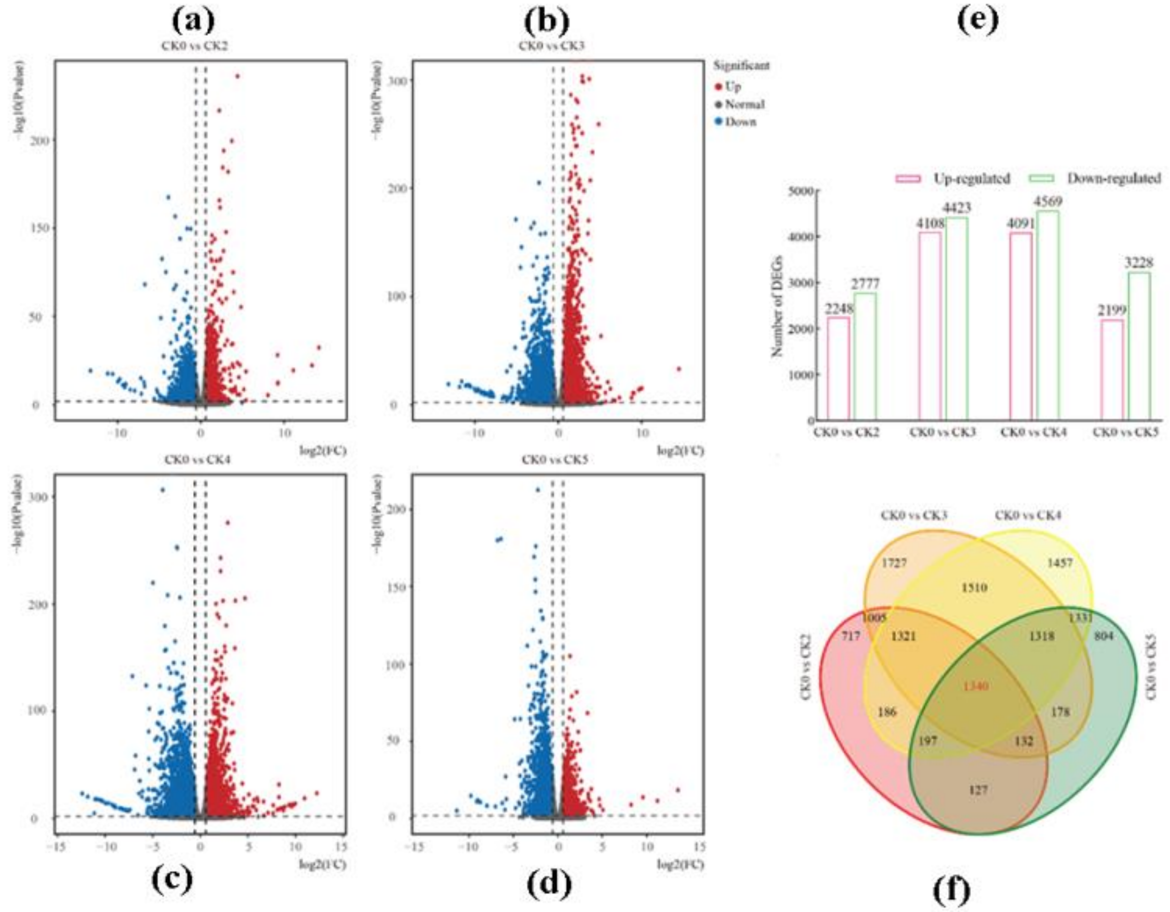

Supplementary Figure S1: Differential gene expression distribution in flower spikes in control groups under low-temperature conditions. Here, (**a**–**d**) indicate differential expression volcano plots comparison among CK0 (0d) vs CK2 (2d), CK0 (0d) vs CK3 (3d), CK0 (0d) vs CK4 (4d), and CK0 (0d) vs CK5 (5d), respectively. Where (**e**) indicates the histogram, and (**f**) indicates the Venn diagram of the number of DEGs in the CK groups.

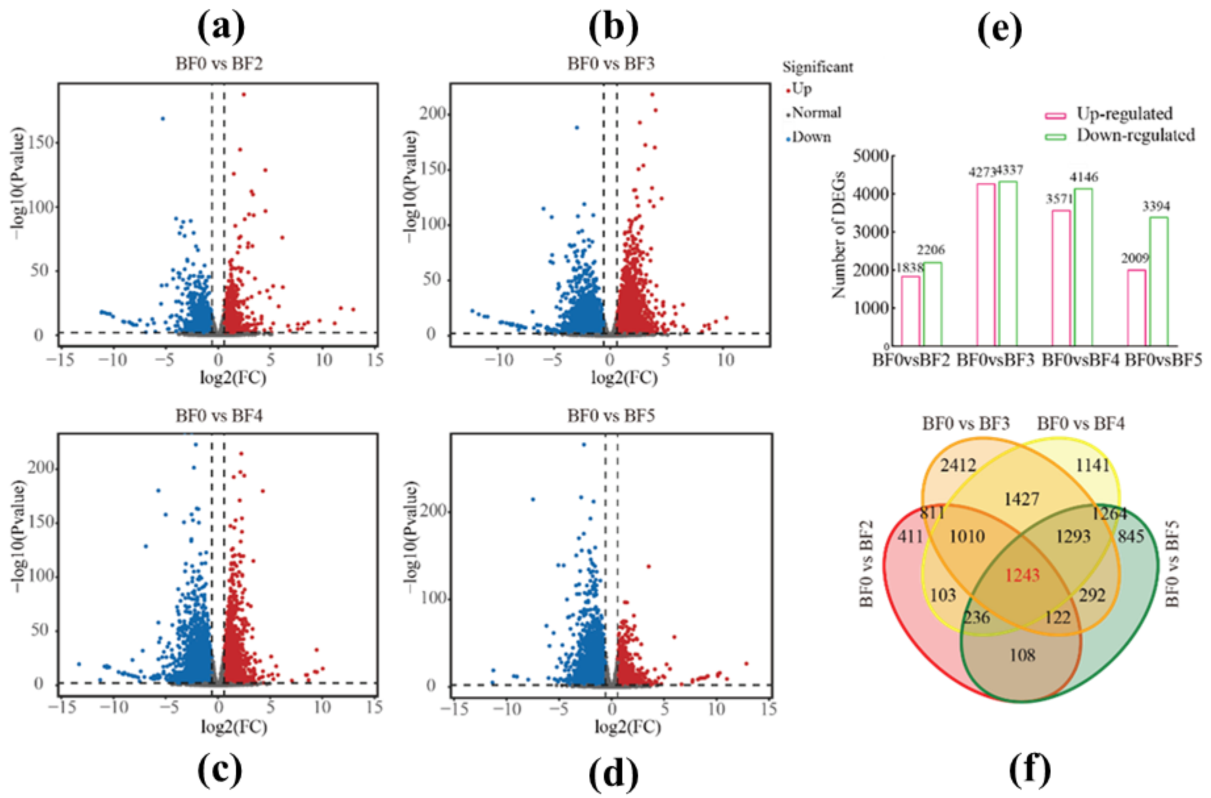

Supplementary Figure S2: Differential gene expression distribution in flower spikes under low temperature conditions in Bihu treatment groups. Here, (**a**–**d**) indicate differential expression volcano plots comparison among BF0 (0d) vs BF2 (2d), BF0 (0d) vs BF3 (3d), BF0 (0d) vs BF4 (4d), and BF0 (0d) vs BF5 (5d), respectively. Where (**e**) indicates the histogram, and (**f**) indicates the Venn diagram of the number of DEGs in the BF groups.

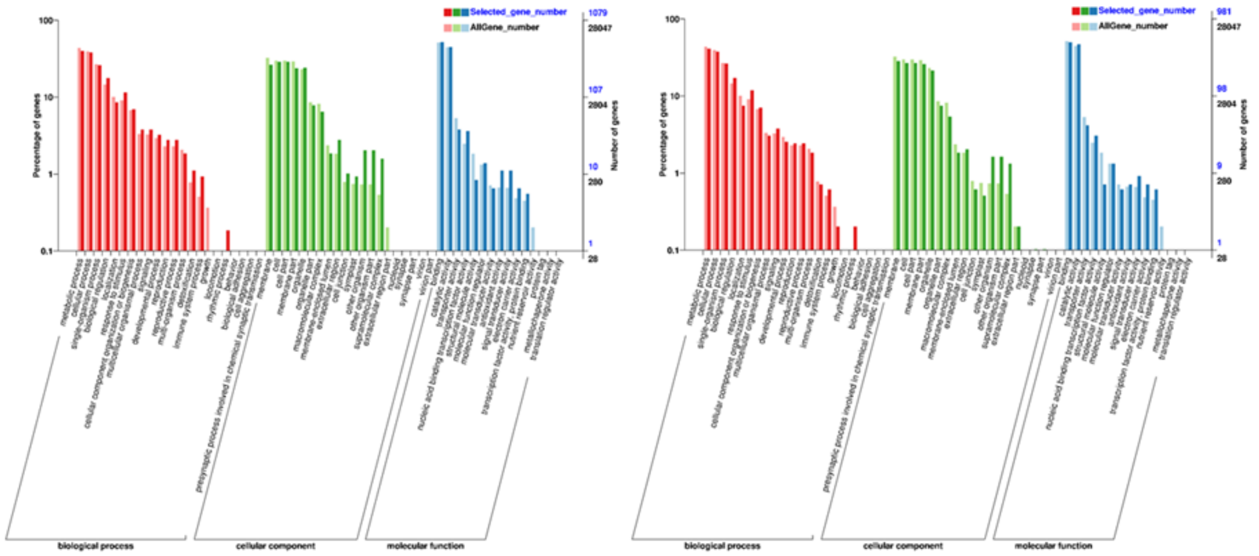

(a) (b)

Supplementary Figure S3: GO classification map of low-temperature response genes in flower spikes. Here, (**a**) indicates the GO classification map in CK groups, and (**b**) indicates the GO classification map in BF groups.

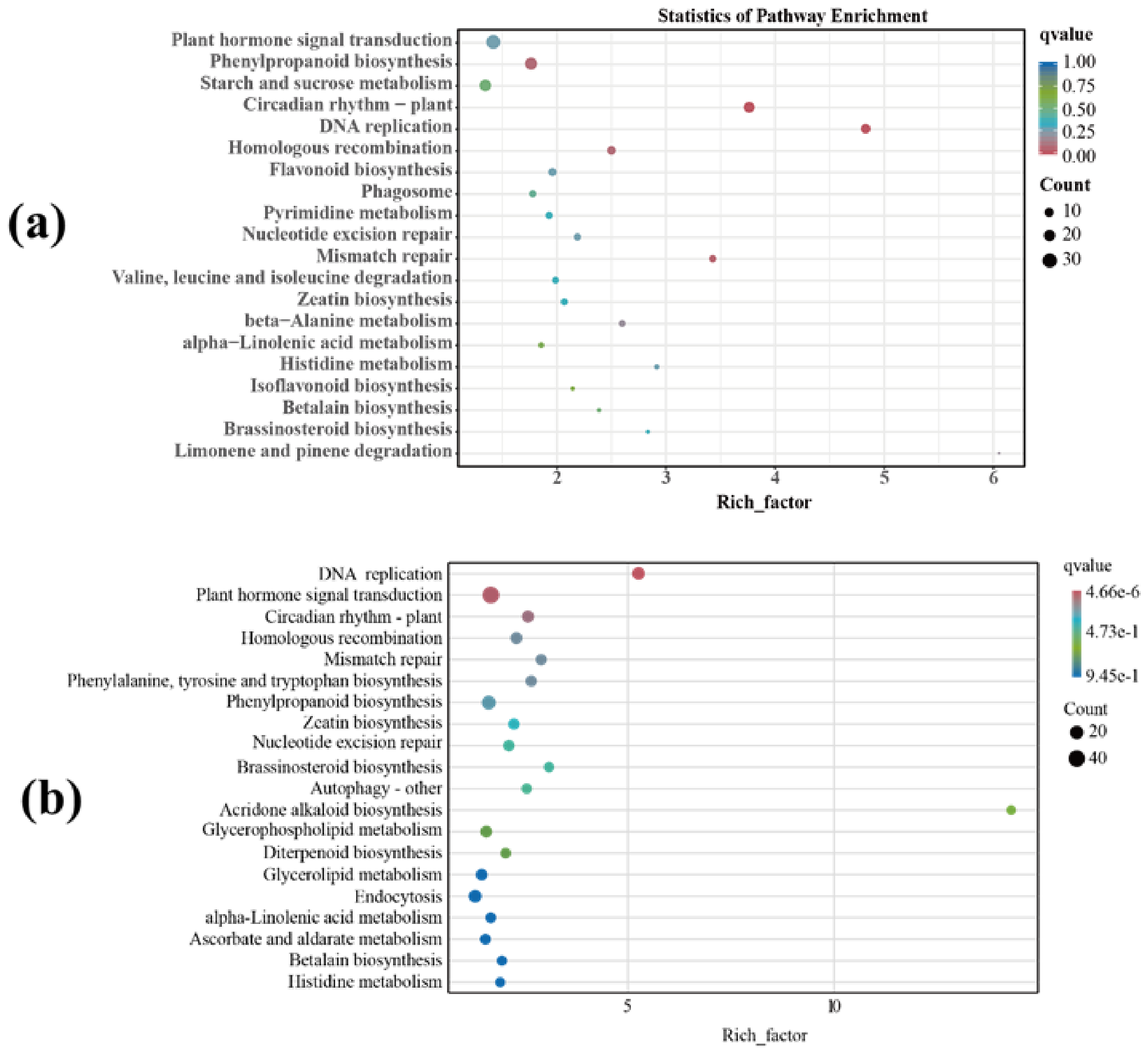

Supplementary Figure S4: KEGG classification map of low-temperature response genes in flower spikes. Here, (**a**) indicates the KEGG classification map in CK groups, and (**b**) indicates the KEGG classification map in BF groups.

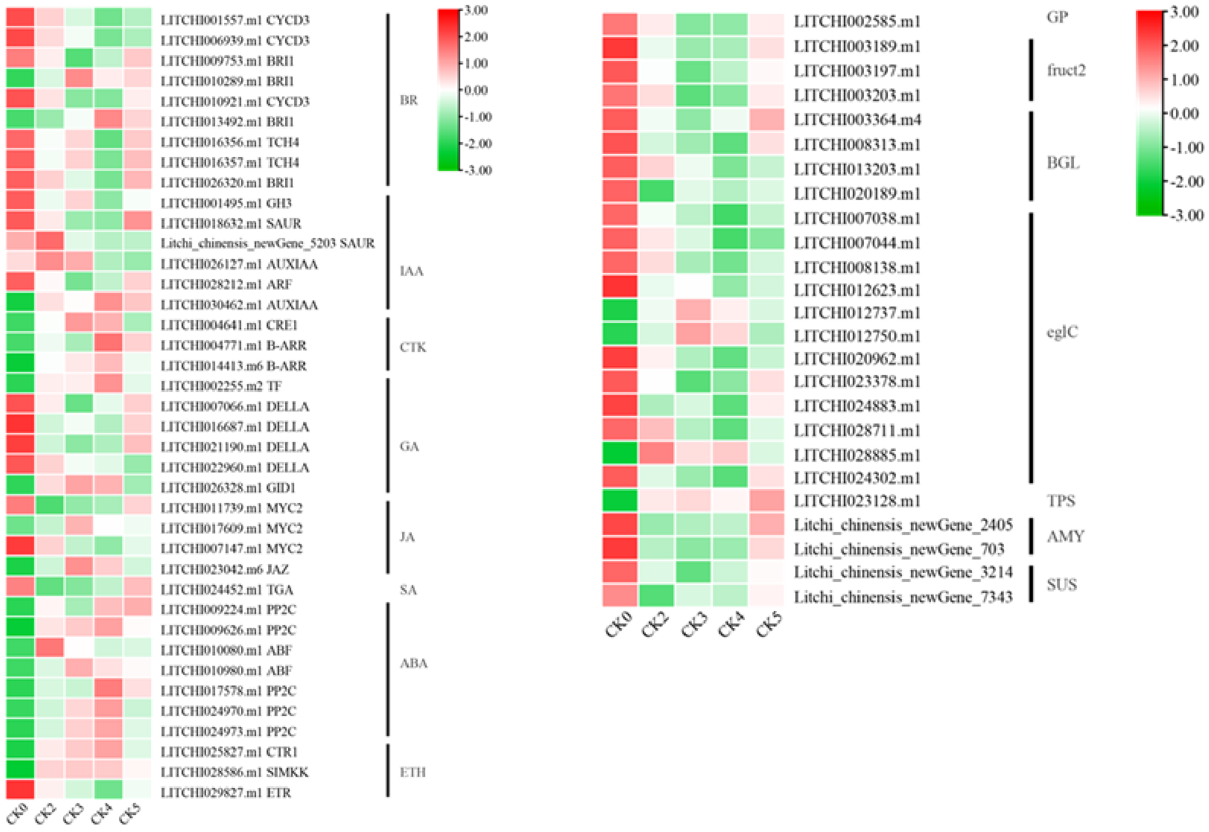

(a) **(b)**

Supplementary Figure S5: Heat map of plant growth regulator candidate gene enrichment pathways in CK groups under low temperature. Here, (**a**) indicates plant hormone signal transduction and (**b**) indicates starch and sucrose metabolism.

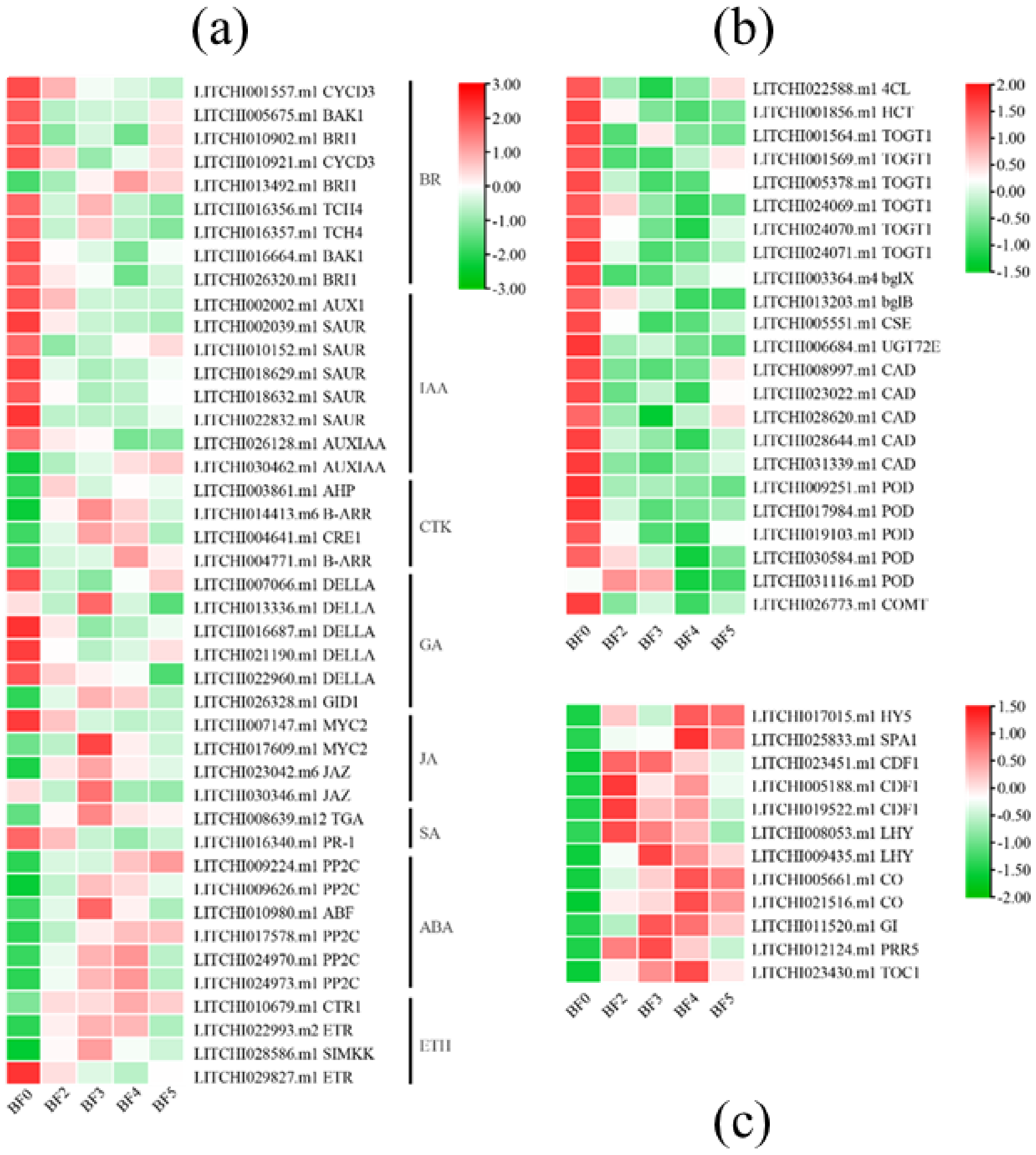

Supplementary Figure S6: Heat map of plant growth regulator candidate gene enrichment pathways in BF groups under low temperature. Here, (**a**) indicates a heat map of growth regulator candidate genes enriched to plants' hormone signal transduction, (**b**) indicates a heat map of growth regulator candidate genes enriched to phenylpropanoid biosynthesis, and (**c**) indicates a heat map of growth regulator candidate genes enriched to the circadian rhythm-plant pathway.

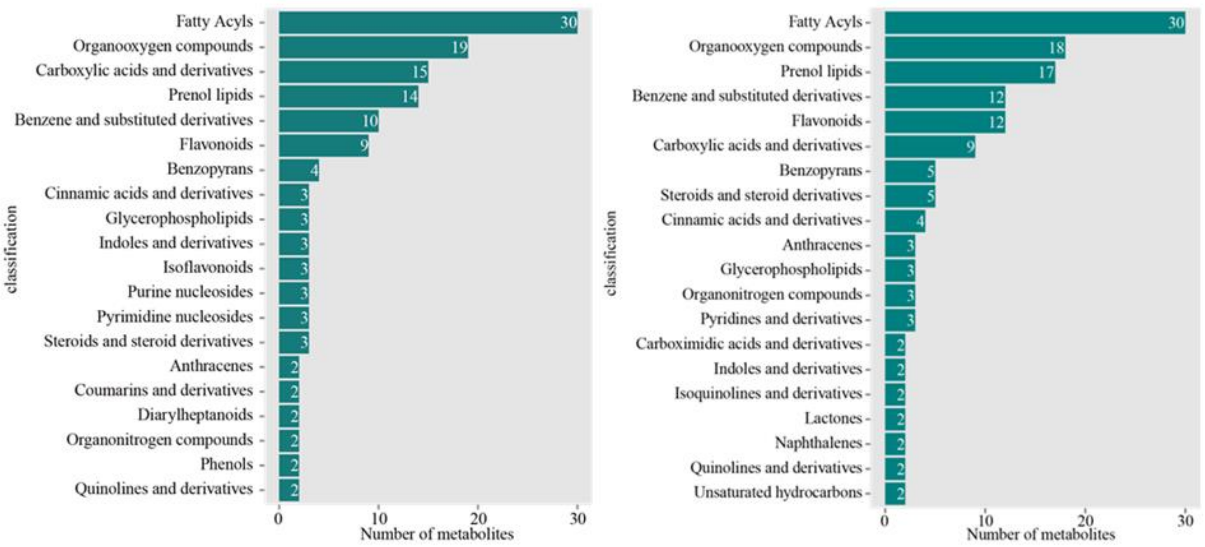

(a) **(b)**

Supplementary Figure S7: Top 20 classification chart of metabolites in flower spikes under low-temperature conditions. Here, (**a**) indicates CK groups, and (**b**) indicates BF groups of the top 20 classification chart of metabolites.

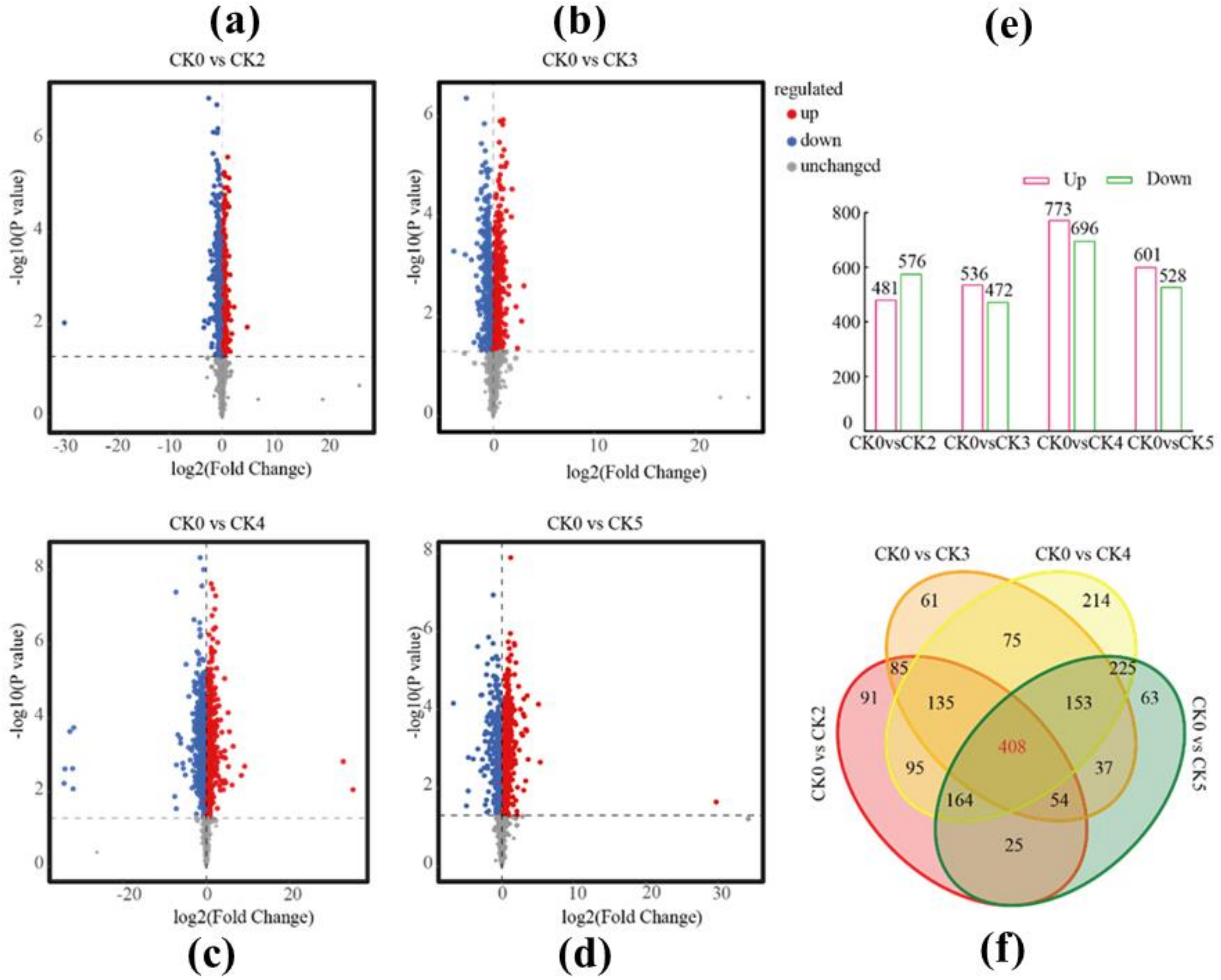

Supplementary Figure S8: Differential Metabolite content distribution of flower spikes under low temperature conditions. Here, (**a–d**) indicate differential expression volcano plots comparison among CK0 (0d) vs CK2 (2d), CK0 (0d) vs CK3 (3d), CK0 (0d) vs CK4 (4d), and CK0 (0d) vs CK5 (5d), respectively. Where (**e**) indicate, the histogram, and (**f**) indicates the Venn diagram of the number of DEMs in the CK groups.

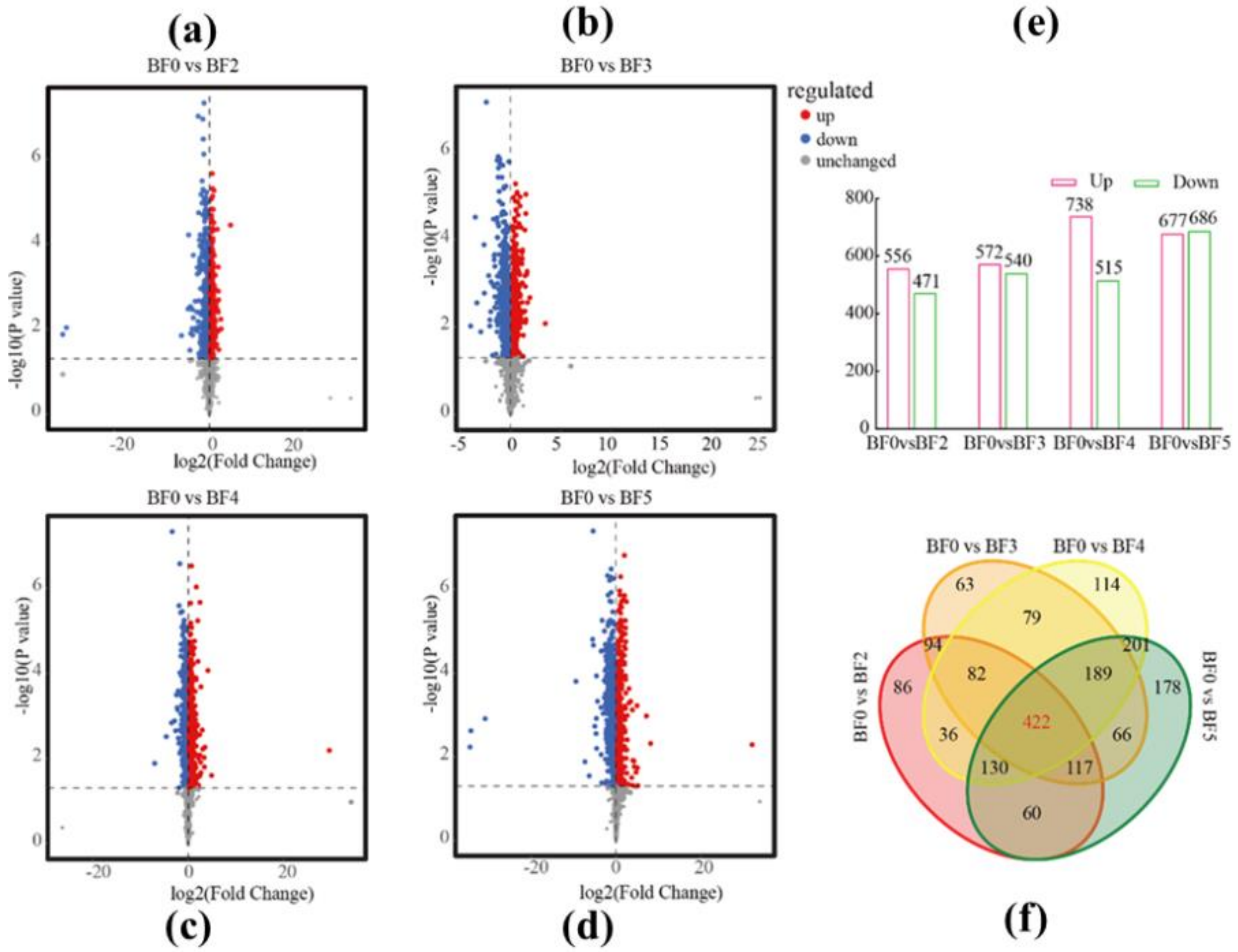

Supplementary Figure S9: Differential metabolites of flower spikes under low temperature conditions treated with plant growth regulators. Here, (**a**–**d**) indicate differential volcano plots comparison among BF0 (0d) vs BF2 (2d), BF0 (0d) vs BF3 (3d), BF0 (0d) vs BF4 (4d), and BF0 (0d) vs BF5 (5d), respectively. Where (**e**) indicates the histogram, and (**f**) indicates the Venn diagram of the number of DEMs in the BF groups.

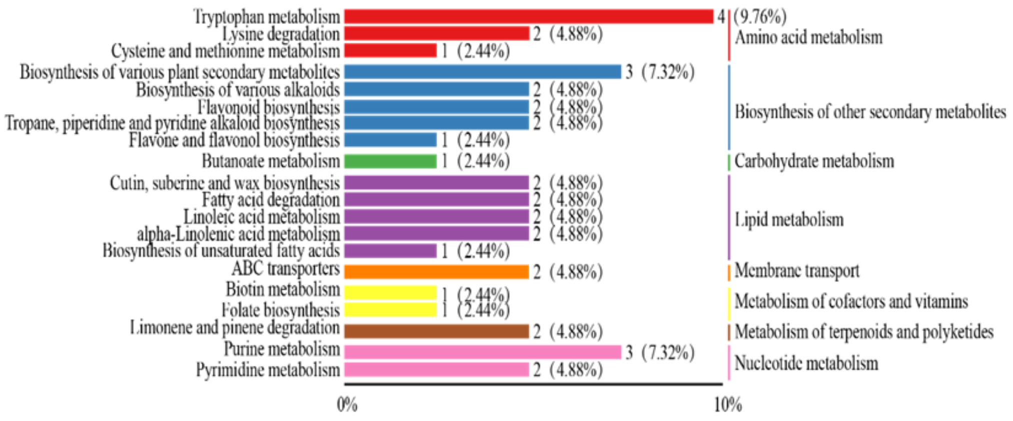

Supplementary Figure S10: Metabolite content distribution of flower spikes under low temperature conditions.

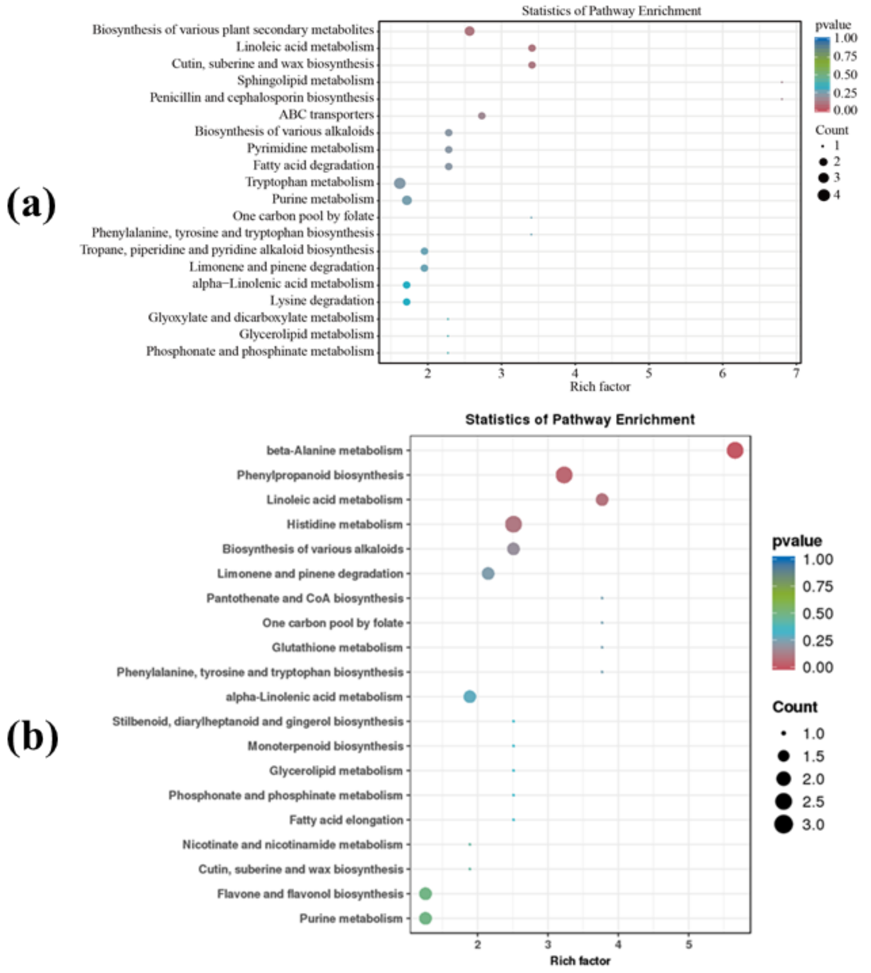

Supplementary Figure S11: KEGG bubble plots of metabolites in flower spikes under low temperature conditions. Here, (**a**) indicates CK groups, and (**b**) indicates BF groups.

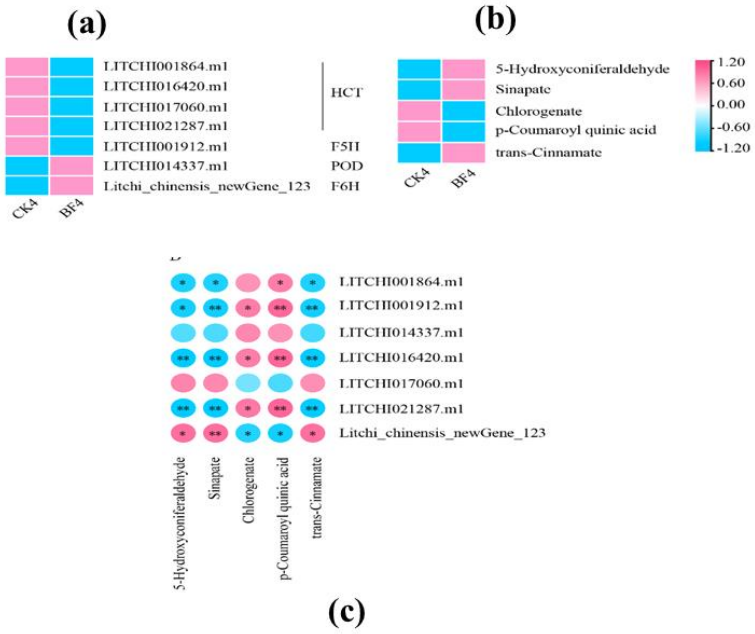

Supplementary Figure 12: Correlation analysis of thermal map of DEGs and DAMs for phenylpropyl biosynthesis pathway**.** Here, (**a**) Thermogram of phenyl propyl biological pathway DEGs, (**b**) Thermal map of the phenyl propyl biological pathway DAMs, (**c**) The correlation heat map of phenyl propyl biosynthesis path DEGs and DAMs shows that no star indicators no significant correlation ($p > 0.05$), one-star indicators significant correlation ($p < 0.05$), and two-star circles indicate very significant correlation ($p < 0.01$).

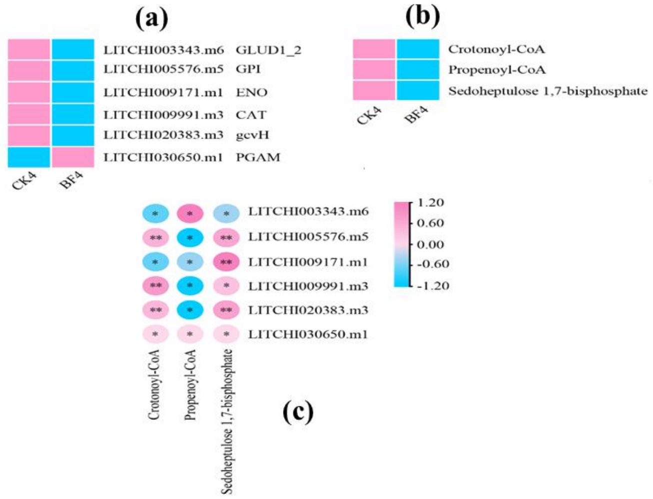

Supplementary Figure S13: Correlation analysis of the thermal map of DEGs and DAMs for Carbon metabolism pathway. Here, (**a**) a Thermogram of the Carbon metabolism

pathway DEGs, (**b**) a thermal map of the Carbon metabolism pathway DAMs, **(c)** The correlation heat map of the Carbon metabolism pathway DEGs and DAMs.

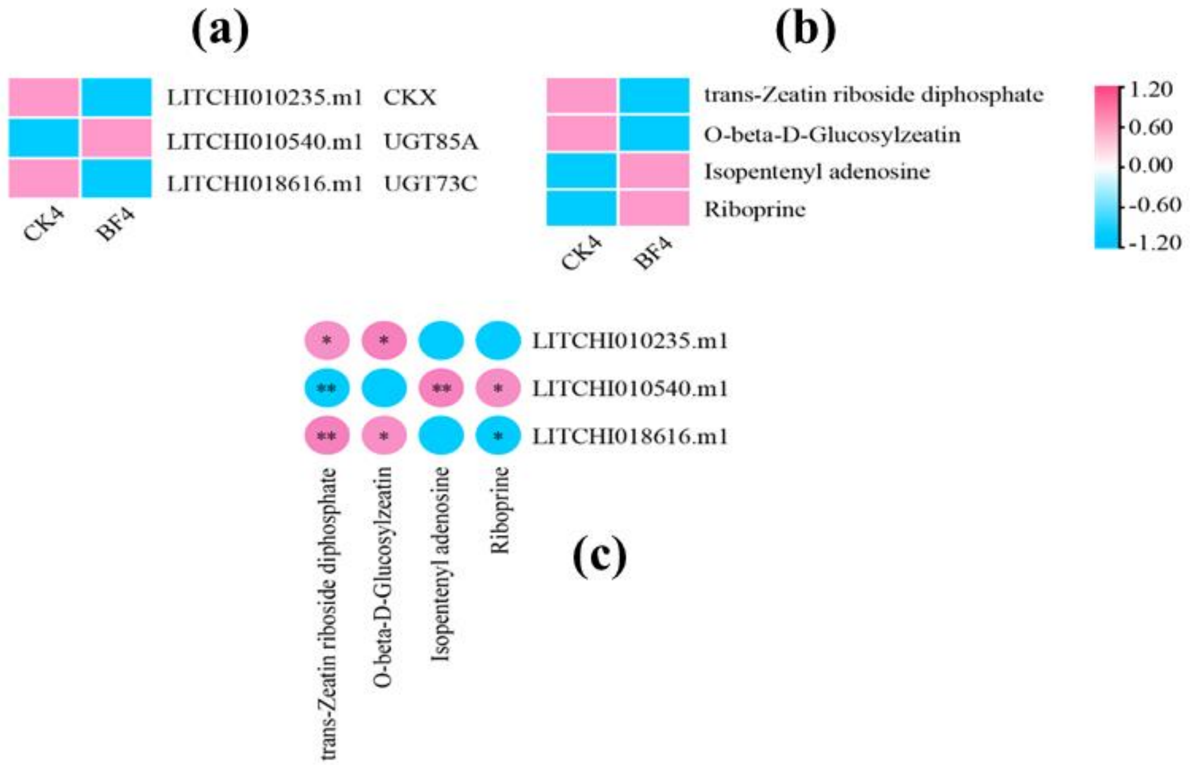

Supplementary Figure S14: Correlation analysis of thermal map of DEGs and DAMs for Zeatin biosynthesis pathway**.** Here, (**a**,**b**) indicate the heat map of zeatin biosynthetic pathway DEGs and DAMs, respectively, (**c**) the Correlation heat map of zeatin biosynthetic pathway DEGs and DAMs.

Supplementary Tables

Supplementary Table S1 : Fruit Survival Percentage (%).

	Fruit Survival Percentage (%) (Mean ± SD)		
**Treatment**	**1.5 Month**	**2.5 Month**	**3.0 Month**
**CK**	15.42 ± 6.09	12.42 ± 6.93	8.58 ± 8.44
**BF**	51.92 ± 5.09	46.92 ± 5.23	30.08 ± 6.50
**LL**	31.25 ± 2.19	26.25 ± 3.59	23.33 ± 3.7

Supplementary Table S2. SOD, POD, CAT activity, and MDA content.

**SOD Activity nmol^{-1}(mg protein)$^{-1}$ (Mean ± SD)**							
**Treatment**	**0 Day**	**2day**	**3day**	**4 day**	**5 day**	**8 day**	**14 day**
**CK**	2.88 ± 0.03 ^{Ad}	5.35 ± 0.22 ^{Aa}	4.18 ± 0.16 ^{Ab}	2.03 ± 0.18 ^{Be}	1.47 ± 0.09 ^{ABf}	2.41 ± 0.06 ^{Ae}	3.39 ± 0.11 ^{Ac}
**BF**	2.88 ± 0.03 ^{Ac}	1.38 ± 0.09 ^{Ce}	4.32 ± 0.14 ^{Aa}	4.41 ± 0.22 ^{Aa}	1.82 ± 0.13 ^{Ad}	2.12 ± 0.11 ^{ABd}	3.63 ± 0.04 ^{Ab}
**LL**	2.88 ± 0.03 ^{Aa}	2.90 ± 0.03 ^{Ba}	1.78±0.05 ^{Bc}	2.19 ± 0.11 ^{Bb}	1.42 ± 0.05 ^{Bd}	1.96 ± 0.08 ^{Bc}	2.28 ± 0.015 ^{Bb}
**POD activity nmol^{-1}(mg protein)$^{-1}$ (Mean ± SD)**							
**CK**	3.52 ± 0.026 ^{Acd}	3.77 ± 0.006 ^{Abcd}	4.82 ± 0.043 ^{Aab}	4.36 ± 0.032 ^{Babc}	3.18 ± 0.036 ^{Bd}	6.08 ± 0.049 ^{Aa}	4.26 ± O.040 ^{Aabcd}
**BF**	3.52 ± 0.026 ^{Ad}	3.45 ± 0.008 ^{Bd}	3.21 ± 0.037 ^{Be}	4.90 ± 0.046 ^{Aa}	4.32 ± 0.038 ^{Ab}	4.01 ± 0.038 ^{Ac}	3.18 ± 0.026 ^{Ce}
**LL**	3.52 ± 0.026 ^{Ad}	3.33 ± 0.02 ^{Ce}	2.55 ± 0.024 ^{Cg}	3.09 ± 0.032 ^{Cf}	4.24 ± 0.029 ^{Ab}	4.80 ± 0.090 ^{Aa}	3.73 ± 0.012 ^{Bc}
**CAT activity nmol^{-1}(mg protein)$^{-1}$ (Mean ± SD)**							
**CK**	1.97 ± 0.86 ^{Aab}	1.61 ± 0.15 ^{Ab}	2.13 ± 0.12 ^{Ba}	1.10 ± 0.18 ^{Ac}	0.33 ± 0.08 ^{Bd}	0.61 ± 0.13 ^{ABd}	0.53 ± 0.07 ^{Bd}
**BF**	1.97 ± 0.867 ^{Ac}	0.35 ± 0.06 ^{Be}	2.80 ± 0.17 ^{Ab}	3.34 ± 0.20 ^{Ba}	1.21 ± 0.15 ^{Ad}	0.41 ± 0.35 ^{Be}	1.40 ± 0.08 ^{Ad}
**LL**	1.97 ± 0.867 ^{Aa}	0.56 ± 0.06 ^{Bd}	1.08 ± 0.04 ^{Cc}	1.01 ± 0.07 ^{Bc}	0.52 ± 0.10 ^{Bd}	0.82 ± 0.12 ^{Acd}	1.47 ± 0.13 ^{Ab}
**MDA activity (nmol.g^{-1} FW) (Mean ± SD)**							
**CK**	0.70 ± 0.05 ^{Ab}	0.83 ± 0.03 ^{ABa}	0.88 ± 0.00 ^{Aa}	0.89 ± 0.03 ^{Aa}	0.67 ± 0.01 ^{Ab}	0.55 ± 0.01 ^{Bc}	0.68 ± 0.03 ^{Bb}
**BF**	0.70 ± 0.05 ^{Ac}	0.85 ± 0.05 ^{Aab}	0.91 ± 0.01 ^{Aa}	0.74 ± 0.04 ^{Bbc}	0.72 ± 0.02 ^{Ac}	0.75 ± 0.02 ^{Abc}	0.71 ± 0.02A ^{ABc}
**LL**	0.70 ± 0.05 ^{Abc}	0.68 ± 0.05 ^{Bc}	0.90 ± 0.02 ^{Aa}	0.81 ± 0.02 ^{ABab}	0.67 ± 0.06 ^{Ac}	0.72 ± 0.01 ^{Abc}	0.77 ± 0.02 ^{Abc}

Supplementary Table S3: Soluble Protein and Proline content.

**Soluble Protein (mg/g) (Mean ± SD)**							
**Treatment**	**0 Day**	**2day**	**3day**	**4 day**	**5 day**	**8 day**	**14 day**
**CK**	14.34 ± 0.23 ^{Ac}	16.34 ± 0.32 ^{Ab}	16.28 ± 0.11 ^{Bb}	12.34 ± 0.28 ^{Bd}	11.23 ± 0.10 ^{Be}	6.95 ± 0.16 ^{Cf}	17.68 ± 0.24 ^{Aa}
**BF**	14.34 ± 0.23 ^{Ad}	9.79 ± 0.02 ^{Bf}	17.01 ± 0.13 ^{Aa}	16.33 ± 0.21 ^{Ab}	17.44 ± 0.26 ^{Aa}	11.17 ± 0.20 ^{Be}	15.54 ± 0.11 ^{Bc}
**LL**	14.34 ± 0.23 ^{Ac}	9.25 ± 0.04 ^{Bf}	9.99 ± 0.06 ^{Ce}	8.42 ± 0.04 ^{Cg}	11.71 ± 0.26 ^{Bd}	16.67 ± 0.27 ^{Aa}	15.13 ± 0.17 ^{Bb}
**Proline content (ug/g) (Mean ± SD)**							
**CK**	116.82 ± 9.88 ^{Ad}	197.57 ± 17.36 ^{Ac}	131.28 ± 8.85 ^{Ad}	221.33 ± 3.62 ^{Cc}	306.04 ± 10.35 ^{Ab}	343.75 ± 13.08 ^{Aa}	194.82 ± 7.63 ^{Ac}
**BF**	116.82 ± 9.88 ^{Ad}	88.76 ± 5.47 ^{Bd}	119.06 ± 11.94 ^{Ad}	422.78 ± 11.62 ^{Aa}	284.18 ± 35.47 ^{Ab}	185.69 ± 19.20 ^{Bc}	253.53 ± 30.23 ^{Ab}
**LL**	116.82 ± 9.88 ^{Acd}	96.16 ± 8.69 ^{Bd}	138.52 ± 8.66 ^{Ac}	321.88 ± 21.9 ^{Ba}	267.3 ± 12.53 ^{Ab}	98.23 ± 3.13 ^{Cd}	259.56 ± 10.13 ^{Ab}

Supplementary Table S4: Fructose, glucose, sucrose, and total sugar content.

Treatment	0 Day	2day	3day	4 day	5 day	8 day	14 day
**Fructose content (mg/g) (Mean ± SD)**							
**CK**	1.15 ± 0.004 ^{Aab}	0.70 ± 0.003 ^{Ae}	0.77 ± 0.009 ^{Ac}	1.14 ± 0.009 ^{Bb}	1.17 ± 0.004 ^{Ca}	0.67 ± 0.007 ^{Bf}	0.74 ± 0.009 ^{Bd}
**BF**	1.15 ± 0.004 ^{Ac}	0.41 ± 0.009 ^{Cg}	0.71 ± 0.01 ^{Bf}	1.28 ± 0.005 ^{Aa}	1.25 ± 0.002 ^{Bb}	1.05 ± 0.025 ^{Ad}	0.84 ± 0.013 ^{Ae}
**LL**	1.15 ± 0.004 ^{Ab}	0.58 ± 0.002 ^{Bg}	0.70 ± 0.01 ^{Bf}	0.95 ± 0.022 ^{Cd}	1.34 ± 0.001 ^{Aa}	1.08 ± 0.003 ^{Ac}	0.88 ± 0.023 ^{Ae}
**Glucose content (mg/g) (Mean ± SD)**							
**CK**	8.35 ± 0.025 ^{Ac}	7.58 ± 0.022 ^{Ae}	8.45 ± 0.023 ^{Ab}	8.26 ± 0.012 ^{Bd}	10.22 ± 0.024 ^{Aa}	5.38 ± 0.046 ^{Cg}	6.35 ± 0.015 ^{Cf}
**BF**	8.35 ± 0.025 ^{Ab}	4.14 ± 0.067 ^{Cf}	6.81 ± 0.014 ^{Cd}	10.28 ± 0.017 ^{Aa}	8.33 ± 0.008 ^{Bb}	5.92 ± 0.080 ^{Be}	7.27 ± 0.033 ^{Bc}
**LL**	8.35 ± 0.025 ^{Ab}	7.27 ± 0.005 ^{Bd}	7.81 ± 0.114 ^{Bc}	7.8 ± 0.047 ^{Cc}	10.28 ± 0.007 ^{Aa}	6.94 ± 0.009 ^{Ae}	8.38 ± 0.076 ^{Ab}
**Sucrose content (mg/g) (Mean ± SD)**							
**CK**	2.48 ± 0.024 ^{Af}	4.93 ± 0.014 ^{Ad}	7.00 ± 0.011 ^{Ac}	7.75 ± 0.074 ^{Bb}	8.36 ± 0.012 ^{Aa}	4.27 ± 0.037 ^{Ce}	4.18 ± 0.007 ^{Ae}
**BF**	2.48 ± 0.024 ^{Af}	2.77 ± 0.026 ^{Cf}	6.49 ± 0.260 ^{Ac}	9.45 ± 0.151 ^{Aa}	8.69 ± 0.166 ^{Ab}	5.68 ± 0.260$^{B d}$	4.35 ± 0.008 ^{Ae}
**LL**	2.48 ± 0.024 ^{Ae}	4.05 ± 0.015 ^{Bd}	6.61 ± 0.058 ^{Ac}	7.56 ± 0.060 ^{Bb}	8.67 ± 0.135 ^{Aa}	6.47 ± 0.004 ^{Ac}	4.20 ± 0.150 ^{Ad}
**Total Sugar content (mg/g) (Mean ± SD)**							
**CK**	11.99 ± 0.04 ^{Ag}	13.21 ± 0.02 ^{Ad}	16.23 ± 0.01 ^{Ac}	17.16 ± 0.09 ^{Bb}	19.76 ± 0.04 ^{Ba}	10.32 ± 0.09 ^{Cf}	11.26 ± 0.02 ^{Ce}
**BF**	11.99 ± 0.04 ^{Af}	7.33 ± 0.05 ^{Ce}	14.01 ± 0.26^{Cc}	21.02 ± 0.13 ^{Aa}	18.27 ± 0.17 ^{Cb}	12.64 ± 0.30 ^{Bd}	12.46 ± 0.05 ^{Bd}
**LL**	11.99 ± 0.04 ^{Ag}	11.90 ± 0.01 ^{Bf}	15.12 ± 0.09 ^{Bc}	16.32 ± 0.11 ^{Cb}	20.29 ± 0.13 ^{Aa}	14.48 ± 0.01 ^{Ad}	13.46 ± 0.20 ^{Ae}

Supplementary Table S5: Sucrose Phosphate Synthase activity and Sucrose Synthase activity.

Treatment	0 Day	2day	3day	4 day	5 day	8 day	14 day
**Sucrose Phosphate Synthase Activity (umol/s/g FW) (Mean ± SD)**							
**CK**	29.88 ± 1.78 ^{Aa}	14.15 ± 0.34 ^{Bb}	4.69 ± 0.14 ^{Cd}	9.55 ± 0.34 ^{Bc}	6.35 ± 1.51 ^{Bcd}	27.84 ± 2.57 ^{Aa}	7.93 ± 0.17 ^{Ccd}
**BF**	29.88 ± 1.78 ^{Ab}	35.34 ± 2.14 ^{Aa}	8.98 ± 0.07 ^{Bde}	7.27 ± 0.09 ^{Ce}	23.25 ± 2.71 ^{Ac}	5.17 ± 0.77 ^{Be}	12.81 ± 1.19 ^{Bd}
**LL**	29.88 ± 1.78 ^{Aa}	1.83 ± 0.10 ^{Ce}	11.08 ± 0.43 ^{Abc}	13.56 ± 0.68^{Ab}	7.79 ± 0.99 ^{Bd}	9.84 ± 0.40^{Bcd}	31.62 ± 0.62 ^{Aa}
**Sucrose Synthase Activity (umol/s/g FW) (Mean ± SD)**							
**CK**	25.89 ± 1.65 ^{Aa}	15.76 ± 0.88 ^{Bc}	5.21 ± 0.40 ^{Be}	10.48 ± 0.44 ^{Bd}	8.31 ± 1.18^{Bde}	22.73 ± 1.32 ^{Ab}	5.50 ± 0.56 ^{Ce}
**BF**	25.89 ± 1.65 ^{Ac}	51.25 ± 2.62 ^{Aa}	8.06 ± 0.48 ^{Ae}	7.63 ± 0.41 ^{Be}	39.95 ± 2.38 ^{Ab}	7.18 ± 1.08 ^{Be}	13.90 ± 0.79^{Bd}
**LL**	25.89 ± 1.65 ^{Ab}	2.86 ± 0.35 ^{Cf}	9.51 ± 0.38 ^{Ade}	15.76 ± 2.12 ^{Ac}	10.59 ± 1.33 ^{Bd}	5.14 ± 1.33 ^{Bef}	37.61 ± 2.85 ^{Aa}

Supplementary Table S6: P5CS, P5CR, ProDH, and δ- OAT activity.

Treatment	0 Day	2day	3day	4 day	5 day	8 day	14 day
**P5CS activity (μmol/h/g) (Mean)**							
**CK**	1.0637	1.0839	1.0358	1.1385	1.3283	1.1215	1.1419
**BF**	1.0637	1.7561	1.8194	2.2883	2.129	2.0903	1.9031
**LL**	1.0637	2.4146	2.8531	2.2635	2.2251	2.2479	1.9812
**P5CR activity (nmol/min/g) (Mean)**							
**CK**	137.72	136.75	106.46	131.09	106.63	129.45	114.49
**BF**	137.72	92.724	98.206	118.93	159.91	150.55	122.81
**LL**	137.72	107.69	128.07	138	115.2	129.7	134.7

**ProDH activity (U/g) (Mean)**							
**CK**	467.05	464.51	422.07	552.59	366.45	419.81	406.6
**BF**	467.05	467.56	584.2	530.8	435.53	508.46	449.27
**LL**	467.05	597.96	488.99	497.09	390.04	566.71	567.23
**δ- OAT activity (nmol/min/g) (Mean)**							
**CK**	24.067	22.764	27.625	29.636	23.465	18.895	18.182
**BF**	24.067	36.117	36.473	38.674	39.834	41.305	40.952
**LL**	24.067	40.785	42.491	44.536	31.239	33.846	40.073
